# Tuning Connectivity in Hybrid Organic–Inorganic Antimony Halides through Reactant Concentration Effects

**DOI:** 10.1021/acs.inorgchem.6c01714

**Published:** 2026-07-11

**Authors:** Jakob Blahusch, Julia Rauh, Petra Rovo, Igor Moudrakovski, Douglas H. Fabini, Daniel Graf, Christian Ochsenfeld, Bettina V. Lotsch

**Affiliations:** † 28326 Max Planck Institute for Solid State Research, Heisenbergstraße 1, 70569 Stuttgart, Germany; ‡ Department of Chemistry, Ludwig-Maximilians-Universität München, Butenandtstraße 5–13, 81377 München, Germany

## Abstract

We report the synthesis and characterization of two hybrid organic–inorganic (HOI) antimony halides by varying the concentration of Sb_2_O_3_, CuCl_2_, and DABCO (DABCO = 1,4-diazabicyclo[2.2.2]­octane) in concentrated hydrochloric acid. Different concentrations lead to different antimony oxidation states, resulting in different inorganic substructures ranging from isolated units to layers and a three-dimensional metal halide framework. (DABCOH_2_)­(Sb^V^Cl_6_)Cl (**1**) contains isolated [Sb^V^Cl_6_]^−^ units separated by the organic cation and chloride. Mixed-valent (DABCOH_2_)_12_(Sb^III^)_14_(Sb^V^)_2_Cl_79_(H_3_O)_3_(H_2_O)_33_ (**2**) features a three-dimensional framework of [Sb^III^Cl_6_]^3–^ units with a previously unknown network topology as well as [Sb^V^Cl_6_]^−^ and an octahedral chloride hydrate cluster with chloride at the center of the water shell. Compound **2** was characterized by single-crystal X-ray diffraction, spectroscopic, and computational methods. A chloride hydrate cluster is also present in the literature-known (DABCOH_2_)_4_Sb_2_^III^Cu_2_^II^Cl_18_(H_2_O)_4_, obtained at the highest reactant concentrations and characterized here by solid-state NMR. These compounds show that the chemical space of antimony halides can be systematically expanded by careful reaction control.

## Graphical abstract

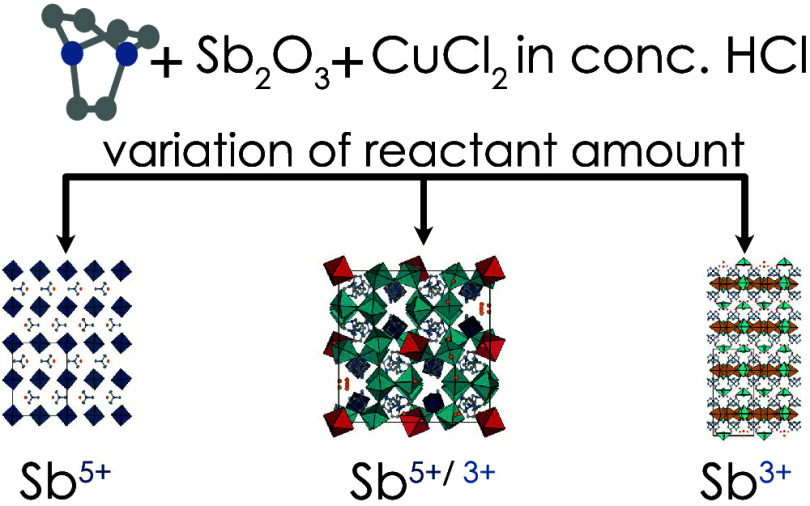

## Introduction

Hybrid organic–inorganic (HOI) metal halides are a growing class of materials combining organic and inorganic characteristics.^1,2^ Among the HOI metal halides, antimony halides have emerged as an interesting class of materials due to the large variety of inorganic building units (isolated units, polyanionic chains, layered structures, and three-dimensional frameworks) as well as different oxidation states of antimony (III, V, or mixed-valent), resulting in physical properties such as ferroelectricity and photoluminescence.^3,4^ While most HOI antimony halides contain isolated units, chains, or layers, Piecha et al. reported the synthesis and structure of [C_3_N_2_H_5_]_6_Sb_4_^III^Br_18_(H_2_O)_2_ (C_3_N_2_H_5_^+^ = pyrazolium), the first hybrid antimony bromide with a three-dimensional framework. This compound marks the first antimony-halide framework material.^5^ The structure is built up of cyclic tetramers and discrete chains linked to each other, thereby forming the framework. Liu et al. reported the synthesis and structure of (C_3_H_9_S)­[Sb^III^Cl_4_] (C_3_H_9_S^+^ = trimethylsulfonium), the first HOI antimony chloride with a three-dimensional framework.^6^ The framework is built up from chains that are linked to each other. Examples of extended HOI antimony iodide frameworks have not been reported so far.

Besides antimony halide materials with antimony in only one oxidation state, mixed-valent antimony halides have been researched since the early 1900s.^7^ Several compounds with the formula A_4_Sb^III^Sb^V^X_12_ (A = Cs^+^, Rb^+^, K^+^, NH4^+^, PEA^+^ = phenylethylammonium) and X = Cl^–^, Br^–^ have been reported in the literature.^8–13^ Besides these, also (C_7_H_13_NH)_4_Sb^III^Sb^V^Br_12_· 2Br_2_ (C_7_H_13_NH = quinuclidinium)^14^ has been reported and all these compounds contain Sb^III^ and Sb^V^ in the ratio 1:1, where the octahedral units are isolated and separated by the cation.

Furthermore, (C_5_H_5_NH)_6_Sb^III^Sb_3_^V^Br_24_ (C_5_H_5_NH = pyridinium)^15^ and Rb_23_Bi_*x*_^III^Sb_7–*x*_^III^Sb_2_^V^Cl_54_ (0 ≤ *x* ≤ 7)^16^ have been prepared, which differ from the typical Sb^III^: Sb^V^ = 1:1 ratio.

Mixed-valent compounds can be classified according to the Robin-Day classification into three classes.^17^ Class I materials have distinct sites for each valence and lack intervalence charge transfer. In class II materials, the units with different valencies also occupy distinct sites, but there is an intervalence charge transfer in the visible range. In class III materials, the valence of the sites cannot be distinguished and the materials show several absorption features in the visible range. All of these mixed-valent HOI antimony halide compounds have a characteristic dark color due to the intervalence Sb^III^–Sb^V^ charge transfer.^17–19^

In contrast to the work on HOI antimony halides, research on halide hydrate clusters is limited. Experimental and crystallographic data have been reported for some chloride hydrate clusters, and they can be categorized by their dimensionality and the number of halides into isolated clusters, chain-like clusters, and layers of halide hydrate clusters. Discrete inorganic anion hydrate clusters are rare, and to the best of our knowledge, only [Cl­(H_2_O)]^−^,^20–22^ [(Cl)_2_(H_2_O)_4_]^2–^,^23^ and [(Cl)_2_(H_2_O)_6_]^2–^ ^24^ have been reported so far. Curnow et al. describe all known halide hydrate clusters in the solid state in their review in 2022.^25^ Halide hydrate clusters contain halide-water and water–water interactions. The halide-water interaction is usually stronger than the water–water interactions. All known chloride hydrate clusters have in common that the chloride anion is positioned at the surface of the cluster. Each chloride-water hydrogen bond reduces the electron density on the oxygen lone pairs involved, thereby weakening any subsequent hydrogen bond to that water molecule. Therefore, to reduce the number of halide-water hydrogen bonds the halide is located on the surface of the cluster, rather than contained within the water shell.^25^ Computational studies of small halide hydrate clusters support the trend of favoring the formation of clusters with the halide position on the surface, rather than inside the water shell.^26^ So far, no chloride hydrate cluster with the halide residing in the center of the water shell has been reported. Furthermore, there is a lack of crystallographic and spectroscopic characterization of halide hydrate clusters.

Here, we report the synthesis of two HOI antimony halides with doubly protonated DABCO (DABCO = 1,4-diazabicyclo[2.2.2]­octane) as the organic cation, namely (DABCOH_2_)­(Sb^V^Cl_6_)Cl (**1**) and (DABCOH_2_)_12_(Sb^III^)_14_(Sb^V^)_2_Cl_79_(H_3_O)_3_(H_2_O)_33_ (**2**). DABCO was selected because its doubly protonated form (DABCOH_2_^2+^) is a rigid, compact cation whose well-defined geometry can template a variety of inorganic chloride substructures, as demonstrated by the previously reported (DABCOH_2_)_2_[Sb_2_^III^Cl_10_]­H_2_O^27^ and (DABCOH_2_)_4_Sb_2_^III^Cu_2_^II^Cl_18_(H_2_O)_4_.^28^ Building on these precedents, we explored whether Sb^V^ and mixed-valent Sb^III^/Sb^V^ compounds could be obtained within the same reaction system, and found that the absolute reactant concentration controls the oxidation state of antimony, leading to the formation of the two new compounds reported here. While compound **1** contains Sb^V^ only, compound **2** is a mixed-valent compound with a three-dimensional framework of [Sb^III^Cl_6_]^3–^, which also features an internal chloride hydrate cluster with chloride residing in the center of the water shell. The formation of the compounds depends on the absolute amounts of the reactants dissolved in a fixed volume of concentrated hydrochloric acid. Compound **2** is an example of a three-dimensional framework of connected antimony chloride octahedra; it is also a mixed-valent antimony halide that contains an internal octahedral chloride hydrate cluster. This unique chloride hydrate cluster was characterized using single-crystal diffraction as well as solid-state NMR spectroscopy, infrared spectroscopy, and quantum chemical NMR calculations. We also used solid-state NMR spectroscopy to characterize the chloride-water structure in the recently reported (DABCOH_2_)_4_Sb_2_^III^Cu_2_^II^Cl_18_(H_2_O)_4_.^28^ These compounds hint at a large chemical space of HOI antimony halides which is still unexplored.

## Experimental Section

### Materials

Starting materials were procured from standard commercial sources and used as received (see Figure S1). Sb_2_^III/V^O_4_ was prepared from Sb_2_^III^O_3_ by heating in an alumina crucible in a muffle furnace with air to 550 °C for 12 h (see SI). All samples were prepared by precipitation from hydrochloric acid.

For the synthesis of compound **1** DABCO (1.00 mmol, 113 mg), Sb_2_^III^O_3_ (0.125 mmol, 36.4 mg) and anhydrous Cu^II^Cl_2_ (0.125 mmol, 16.8 mg) were dissolved in 10 mL conc. hydrochloric acid. The solution was heated to 110 °C for 1 h. A clear yellow solution was obtained, from which upon cooling to room temperature yellow plate-like crystals precipitated. The precipitate was vacuum filtered and dried in air at 60 °C. The yield of compound **1** was 53% based on Sb_2_O_3_.

For the synthesis of compound **2** DABCO (2.00 mmol, 227 mg), Sb_2_^III^O_3_ (0.25 mmol, 72.9 mg) and Cu^II^Cl_2_ (0.25 mmol, 33.6 mg) were dissolved in 10 mL conc. hydrochloric acid. The solution was heated to 110 °C for 1 h. A clear yellow solution was obtained. Upon cooling to room temperature, orange cubic crystals precipitated from the solution. The precipitate was vacuum filtered and dried in air at 60 °C. The yield of compound **2** was 60% based on Sb_2_O_3_. The synthesis can also be performed starting directly with Sb^V^ by heating stoichiometric amounts of DABCO (3.00 mmol, 340 mg), Sb_2_^III^O_3_ (1.50 mmol, 437 mg) and Sb_2_^III/V^O_4_(0.25 mmol, 76.9 mg) in 15 mL conc. hydrochloric acid to 100 °C until a clear solution was obtained, and subsequent crystallization at room temperature. The yield of compound **2** without the usage of CuCl_2_ was 52% based on Sb_2_O_3_.

For the synthesis of (DABCOH_2_)_4_Sb_2_^III^Cu_2_^II^Cl_18_(H_2_O)_4_, DABCO (1.00 mmol, 113 mg), Sb_2_^III^O_3_ (0.50 mmol, 146 mg), and Cu^II^Cl_2_ (1.00 mmol, 134 mg) were dissolved in 10 mL conc. hydrochloric acid. The solution was heated to 110 °C for 1 h. Upon cooling to room temperature, red crystals precipitated from the solution. The precipitate was vacuum filtered and dried in air at 60 °C. The yield of (DABCOH_2_)_4_Sb_2_^III^Cu_2_^II^Cl_18_(H_2_O)_4_ was 58% based on Sb_2_O_3_. (DABCOH_2_)_4_Sb_2_^III^Cu_2_^II^Cl_18_(H_2_O)_4_ can also be obtained with lower amounts of Cu^II^Cl_2_ by following the synthesis of compound **2** and using degassed conc. HCl during the synthesis. Degassed conc. HCl was prepared by purging the solution with argon for 10 min and performing the synthesis under argon atmosphere. The yield of (DABCOH_2_)_4_Sb_2_^III^Cu_2_^II^Cl_18_(H_2_O)_4_ via this method was 34% based on Sb_2_O_3_.

### Single-Crystal X-ray Crystallography (SXRD)

SXRD data of compound **1** were collected at room temperature and at 101 K for compound **2** on a Bruker D8 Venture diffractometer equipped with a rotating anode generator with Mo Kα radiation (λ = 0.71073 Å). The diffraction intensities were integrated using the SAINT software package and a multiscan absorption correction was applied with SADABS.^29^ The crystal structure was solved using intrinsic phasing (SHELXT)^30^ and refined against F^2^ by applying the full-matrix least-squares method (SHELXL)^31^ using the software OLEX2.^32^ Hydrogen atoms at C and N atoms were inserted at idealized positions and using a riding model. Hydrogen atoms on the water molecule were refined using DFIX and DANG instructions. All non-hydrogen nondisordered atoms were refined anisotropically using full-matrix least-squares. Compound **1** features positional disorder of two chlorides. Each of the chloride sites refined as a split position with an occupancy of $\frac{1}{2}$ . Compound **2** features disorder and disordered water molecules. The disordered water molecules were removed from the electron density map using the OLEX2 solvent mask command (see SI for further information on disorder handling). The structure of compound **2** features a disordered [Sb^V^Cl_6_]^−^ unit (Sb4), whose six coordinating Cl atoms are disordered over 18 positions with occupancy fixed to $\frac{1}{3}$ ; this site was treated using PART -1 in SHELXL. One DABCOH_2_^2+^ cation shows enlarged anisotropic displacement parameters indicative of unresolved rotational disorder. A detailed description of the disorder handling is given in the SI. The single-crystal quality and absence of twinning or satellite reflections for compound **2** were confirmed by inspecting calculated precession images along the principal reciprocal lattice directions (see Figures S5–S7). ORTEP (Oak Ridge Thermal-Ellipsoid Plot) drawings for compound **1** and **2** are shown in Figures S2 and S3. Crystallographic data have been deposited with the Cambridge Crystallographic Data Centre: Deposit numbers CCDC-2390400 **1**, CCDC-2390399 **2** 101 K.

### Powder X-ray Diffraction (PXRD)

The X-ray powder patterns were obtained with a Stoe Stadi-P diffractometer in Debye–Scherrer geometry, employing Cu Kα_1_ radiation, a Ge(111) monochromator, and a Mythen 1k detector. All samples were filled into glass capillaries with a diameter of 0.3 mm (Hilgenberg GmbH, Germany). The program TOPAS v6.0 ^33^ was used for Pawley fits^34^ to determine the phase purity. During refinements the lattice parameters were refined.

### Solid-State NMR (ssNMR)

Fast magic-angle spinning solid-state ^1^H, ^13^C, and ^15^N NMR measurements of compound **2** were performed on a Bruker Neo 700 MHz NMR spectrometer equipped with a 1.3 mm (HCN) probe. The spinning frequency was set to 55.55 kHz, and the temperature was regulated to a 20 °C nominal temperature. ^1^H, ^13^C, and ^15^N chemical shifts were referenced to an external sodium trimethylsilylpropanesulfonate signal. Typical 90° pulse lengths for ^1^H, ^13^C, and ^15^N were 1.5 μs, 2.7 μs, and 3.5 μs, respectively. ^1^H, ^13^C, and ^15^N MAS of compound **1** as well as ^35,37^Cl MAS and stationary NMR spectra were obtained on a Bruker Neo 600 MHz using 1.3 mm DVT and 4 mm WVT double resonance probes. Data were fitted using the *ssNake* software (Version 1.4).^35^ For ^1^H NMR, a superposition of Gaussian and Lorentzian line shapes was used. The fitting parameters for the measurements at different temperature can be found in Tables S10–S16. For ^35,37^Cl NMR first *C*_Q_ and η and δ_iso_ obtained from the MAS spectra were further used in the analysis of the stationary spectra (see Tables S5, S7, S17, and S19). The stationary ^35,37^Cl spectra were simulated using a combination of quadrupolar interaction and chemical shift anisotropy by keeping *C*_Q_, *ν*_Q_, and δ_iso_ fixed as determined in the MAS sample and fitting anisotropy Δ and Euler angles between the CSA and EFG tensors as described by Bryce et al. (see Tables S6, S8, S18, and S20).^36^

### Quantum Chemical Calculations

The structure of the cluster model employed in this work for the computation of NMR shielding constants was based on crystallographic data. The extracted structure is centered around the chloride hydrate cluster of interest, is charge neutral, and exclusively contains Sb atoms in the oxidation state III. All calculations were performed with the FermiONs++ program package developed in the Ochsenfeld group.^37–39^

For the structure optimization, the PBEh-3c composite method of the Grimme group was employed.^40^ During this procedure, only the water molecules of the chloride hydrate cluster were allowed to relax, while the remaining atoms were kept fixed at their crystallographic positions. The structure optimization was carried out using the gm3 numerical integration grid^41^ for both the density functional approximation (DFA) exchange-correlation parts and the seminumerical evaluation of the exchange contribution (sn-LinK).^42^ We further employed the resolution-of-the-identity approach for the calculation of the Coulomb contribution (RI-J),^43^ together with the def2-universal-jfit auxiliary basis set.^44^ The final optimized structure of the model used for the NMR calculations is provided as an.xyz file in the SI. For the subsequent NMR calculations, which included the computation of the nuclear quadrupole coupling constants, the range-separated hybrid functional ωB97X^45^ was employed. The atomic-orbital (AO) basis sets utilized for these calculations were the def2-TZVP basis set^46^ for Sb, including the respective effective core potential,^47^ and the pcS-2 basis set^48^ for all other atoms. For the RI-J approximation, the def2-QZVP-RI-JK auxiliary basis set^49^ was employed. Numerical integrations were carried out using the larger gm5 grid^41^ for both the exchange-correlation parts and the sn-LinK part. The self-consistent field convergence criteria in all performed calculations were 10^–6^ au for the change in energy, 10^–6^ for the RMS of the FP-commutator, and 10^–6^ for the RMS of the difference density. Convergence was achieved only when all three criteria were satisfied simultaneously.

### Infrared Spectroscopy

FT-IR spectra were recorded on a Spectrum BX by PerkinElmer equipped with a diamond ATR unit from Smiths Detection. The measurement was performed in the range of 4000–650 cm^–1^.

### Optical Spectroscopy

Diffuse reflectance spectra were obtained with a Cary 5000 by Agilent equipped with an integrating sphere. BaSO_4_ was used as the white standard for measurements.

### Simultaneous Thermal Analysis

STA measurements were obtained with a STA449 F5 Jupiter instrument by Netzsch. Corundum crucibles were filled with the sample. They were heated dynamically with heating rates of 10 K min^–1^ under Ar flow. The data were analyzed with the Netzsch Proteus 61 software package.

Crystal structures were visualized with VESTA.^50^ Bond valence sums were calculated in VESTA using parameters from Brese and O’Keeffe^51^ (*R*_0_ = 2.35 Å, *B* = 0.37 Å for Sb^III^; *R*_0_ = 2.30 Å, *B* = 0.37 Å for Sb^V^) as compiled by Brown.^52^ Data were plotted using python and matplotlib.^53^

## Results and Discussion

The synthesis of all three compounds is based on dissolving DABCO, Sb_2_^III^O_3_ and Cu^II^Cl_2_ in conc. HCl at 110 °C and subsequent precipitation at room temperature. By changing the concentration of the reactants, the oxidation state of antimony in the compounds can be controlled, resulting in the formation of different compounds with distinct inorganic building units (see Figure 1). Compound **1** precipitates as yellow crystals from the solution, while compound **2** precipitates as red-orange, block-like crystals and (DABCOH_2_)_4_Sb_2_^III^Cu_2_^II^Cl_18_(H_2_O)_4_ precipitates as dark red blocks. The latter compound has recently been reported by Liu et al.^28^ and crystallizes isostructurally to (DABCOH_2_)_4_Sb_2_^III^Cd_2_^II^Cl_18_(H_2_O)_4_ as reported by Ben Rhaiem et al.^27^ This points toward the possibility of replacing Cd and Cu with other divalent metals which increases the chemical space for this structure type. A summary of the crystallographic information can be found in Table 1. Phase purity was determined by powder X-ray diffraction (see Figures S9 and S10). Performing the synthesis with hydrobromic acid and hydroiodic acid resulted in the formation of compounds, which have a distinctly different structure and will be discussed in a forthcoming paper.

**Figure 1:**
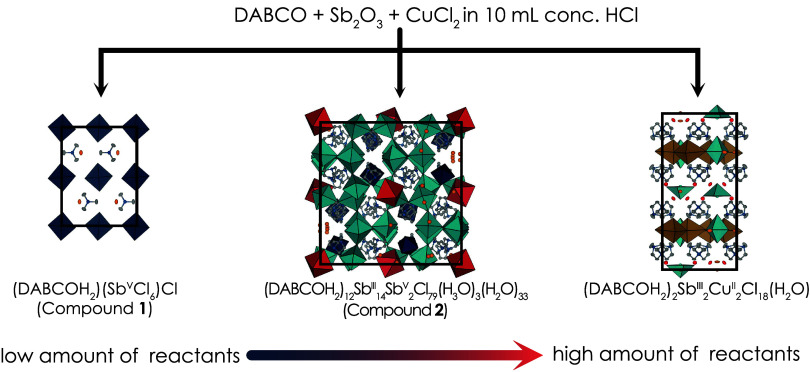
Reaction scheme displaying the formation of compound **1**, **2**, and (DABCOH_2_)_4_Sb_2_^III^Cu_2_^II^Cl_18_(H_2_O)_4_ by increasing the absolute amounts of the reactants dissolved in a fixed volume of concentrated hydrochloric acid. [Sb^V^Cl_6_]^−^ octahedra are shown in dark blue, [Sb^III^Cl_6_]^3–^ octahedra in mint, [Cl­(H_2_O)_6_]^−^ clusters in orange, and [Cu^II^Cl^6^]^4–^ octahedra are shown in brown. Chlorine atoms are shown in orange, nitrogen atoms in light blue, carbon atoms in gray, oxygen atoms in red. Disorder and hydrogen atoms are omitted for clarity.

**Table 1:** Summary of Crystallographic Information for Compounds **1** and **2**

	**1**	**2**
formula	C_6_H_14_Cl_7_N_2_Sb	C_72_H_243_Cl_79_N_24_O_36_Sb_16_
formula weight	484.09	6770.42
temperature, K	298.80	101.90
crystal system	orthorhombic	cubic
space group	*Cmcm* (#63)	*Pa*3̅ (#205)
*a*, Å	7.8054(2)	27.3241(4)
*b*, Å	12.3396(3)	27.3241(4)
*c*, Å	16.4463(4)	27.3241(4)
α, deg	90	90
β, deg	90	90
γ, deg	90	90
volume, Å^3^	1584.03(7)	20400.3(9)
*Z*	4	4
ρ_calc_, g cm^–3^	2.030	2.203
*F*(000)	936.0	13148.0
radiation	Mo Kα (λ = 0.71073 Å)	Mo Kα (λ = 0.71073 Å)
2θ range, deg	6.656–61.046	4.472–61.002
index ranges	–11 ≤ *h* ≤ 11, –17 ≤ *k* ≤ 17, –23 ≤ *l* ≤ 23	–38 ≤ *h* ≤ 39, –39 ≤ *k* ≤ 39, –39 ≤ *l* ≤ 38
reflections collected	14,083	410,459
independent reflections	1321 [*R*_int_ = 0.0353, *R*_σ_ = 0.0172]	10,370 [*R*_int_ = 0.0605, *R*_σ_ = 0.0157]
data/restraints/parameters	1321/0/57	10,370/2/361
goodness-of-fit	1.112	1.126
final *R* indexes [*I* ≥ 2σ(*I*)]	*R*_1_ = 0.0210, *wR*_2_ = 0.0467	*R*_1_ = 0.0489, *wR*_2_ = 0.1060
final *R* indexes [all data]	*R*_1_ = 0.0331, *wR*_2_ = 0.0561	*R*_1_ = 0.0751, *wR*_2_ = 0.1308
largest diff. peak/hole, e Å^–3^	0.59/–0.51	1.85/–1.15

### Reaction Pathway

As shown in Figure 1, the reaction starts with the addition of Cu^II^Cl_2_ to the Sb^III^ precursor, leading to the formation of compound **1** with Sb^V^ only. If the amount of all reactants is increased, the mixed-valent compound **2** is obtained, while with a further increase of Sb_2_^III^O_3_ and Cu^II^Cl_2_ (DABCOH_2_)_4_Sb_2_^III^Cu_2_^II^Cl_18_(H_2_O)_4_ is formed. The concentration of the reactants was varied by scaling the absolute amounts of all reactants proportionally while keeping the solvent volume constant at 10 mL of concentrated hydrochloric acid; the exact amounts used for each compound are summarized in Table S2. This implies the formation of compounds with different oxidation states depending on the concentration of the starting materials. In fact, the oxidation of Sb^III^ to Sb^V^ under acidic conditions has been reported with vanadium­(V),^54^ gold­(III),^55^ and cerium­(IV)^56^ as the oxidation agents. The photooxidation of Sb^III^ to Sb^V^ in CHCl_3_ solution in the presence of oxygen has been reported by Vogler et al. in 1989.^57^ We thus infer that Cu^II^Cl_2_ acts as an oxidant for Sb^III^ to Sb^V^. To determine the potential role of oxygen for the formation of **2**, the synthesis was performed under deaerated conditions, i.e., with degassed conc. HCl was used under an inert gas atmosphere. This resulted in the direct formation of (DABCOH_2_)_4_Sb_2_^III^Cu_2_^II^Cl_18_(H_2_O)_4_ and suggests an oxidation process that involves oxygen in the solution acting as the primary oxidant (see Figure S11). Without any Cu^II^Cl_2_ in the reaction mixture under otherwise similar conditions, (C_6_H_14_N_2_)_2_[Sb_2_^III^Cl_10_]­H_2_O forms as reported by Ben Rhaiem et al.^27^ In fact, without Cu^II^Cl_2_ or without oxygen, the formation of a Sb^V^ compound has not been observed. Therefore, the oxidation of Sb^III^ to Sb^V^ seems to result from an interplay of Cu^II^Cl_2_ and oxygen in the reaction mixture.

### Structural Description

Compound **1** was obtained as yellow crystals and crystallizes in the orthorhombic space group *Cmcm* (#63). The structure can be described as isolated [Sb^V^Cl_6_]^−^ octahedra separated by doubly protonated DABCOnium cations and chloride anions (see Figure 2). The Sb–Cl bond lengths are in the range of 2.337(4) to 2.391(2) Å, suggesting the oxidation state +5 for antimony in accordance with the bond valence sum of 5.196.^13,58–60^ The FT-IR spectrum and solid-state ^1^H, ^13^C, and ^15^N NMR spectra can be found in the SI (see Figures S12–S14 and S32). The octahedral bond-length distortion (Δ_octa_) has been calculated according to the following equation:^61^

1 $$\Delta_{\mathrm{octa}}=\frac{1}{6}\sum_{i=1}^{6}{\left(\frac{d_{i}-d_{\mathrm{av}}}{d_{\mathrm{av}}}\right)}^{2}$$

where *d*_*i*_ is the bond length and *d*_av_ is the average bond length in the octahedron. Δ_octa_ = 0.115 × 10^–3^ and a mean cis–Cl–Sb-Cl of 90°, which is expected for Sb^V^ compounds due to the absence of the stereoactive 5s^2^ lone pair, in contrast to compounds with Sb^III^ (see Table 2).

**Figure 2:**
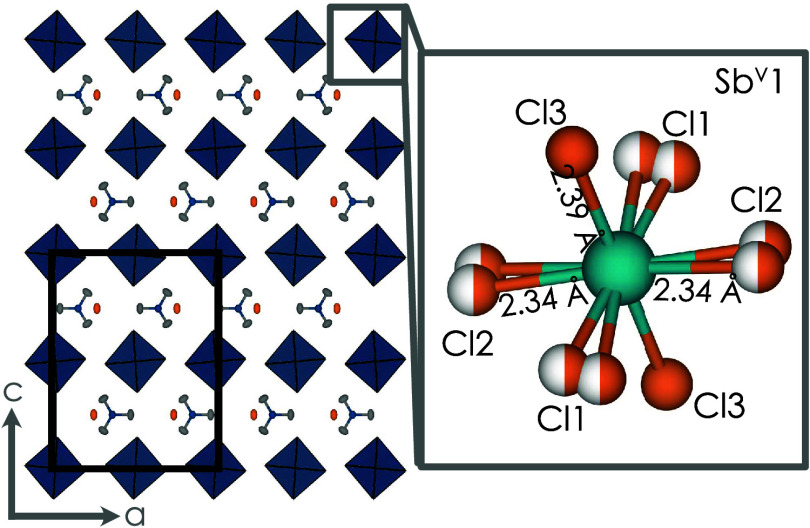
2 × 2 × 2 supercell of the crystal structure of compound **1**, viewed along [010], along with the bond lengths in the [Sb^V^Cl_6_]^−^ octahedra (close-up). [Sb^V^Cl_6_]^−^ octahedra are shown in blue. Chlorine atoms are shown in orange, nitrogen atoms in light blue, carbon atoms in gray, and hydrogen atoms are omitted for clarity. Positional disorder is omitted in the supercell for clarity. Anisotropic displacement ellipsoids are drawn at 50% probability level.

**Table 2:** Octahedral Bond-Length Distortions and Mean Cis-Cl–Sb–Cl Angles in Compounds **1** and **2**

compound	site	Δ_octa_ [10^–3^]	mean cis-Cl–Sb–Cl angles [deg]
**1**	Sb^V^	0.115	90
**2**	Sb^III^(1)	2.32	89.96
	Sb^III^(2)	9.85	91.34
	Sb^III^(3)	6.53	88.23
	Sb^V^(4)	-	-

Increasing the amounts of the reactants during the synthesis results in the precipitation of block-like red-orange crystals of compound **2** which crystallize in the cubic space group *Pa*3̅ (#205) with four formula units per unit cell. **2** can also directly be synthesized by precipitation from hydrochloric acid with stoichiometric amounts of DABCO, Sb_2_^III^O_3_ and Sb_2_^III/V^O_4_ (see Experimental Section). The structure can be described as a three-dimensional framework of corner-sharing [Sb^III^Cl_6_]^3–^ octahedra enclosing doubly protonated DABCOnium cations, disordered [Sb^V^Cl_6_]^−^ units, as well as [Cl­(H_2_O)_6_]^−^ units and disordered crystal water with hydronium ions (see Figure 3). The bond lengths in the [Sb^III^Cl_6_]^3–^ units are in the range of 2.414(3)–2.962(4) Å for terminal Sb–Cl bonds and 2.915(3)–3.024(3) Å for the connecting Sb–Cl bonds. These bond lengths indicate the oxidation state +3 for [Sb^III^Cl_6_]^3–^ units, which is in good agreement with the calculated bond valence sums (2.859, 2.969, 2.876).^27,62^ The octahedra are heavily distorted, as can be seen by the bond-length distortion and deviation of the cis–Cl–Sb-Cl angle from 90° in Table 2. This is consistent with the stereochemically active lone pair of trivalent antimony. In the case of isolated [Sb^III^Cl_6_]^3–^ units a distortion of Δ_octa_ = 2.39 × 10^–3^ has been reported,^63^ for bioctahedral [Sb_2_^III^Cl_10_]^4–^ units Δ_octa_ = 9.95 × 10^–3^,^64^ for chain-like antimony chloride units Δ_octa_ = 11.78 × 10^–3^,^63^ and for a layered structure Δ_octa_ = 15.0 × 10^–3^.^65^ The octahedral distortion not only depends on the connectivity of the inorganic units but also on the environment, and thus these values are only exemplary to show that the distortion in the framework of compound **2** is in the expected range. Several metal halide framework materials have been reported with DABCO as the organic template, but compound **2** is the first example of a DABCO framework material based on antimony chloride units.^66–71^ [Sb_1_Cl_6_]^3–^ units are connected via corners to three [Sb_2_Cl_6_]^3–^ units which can be viewed as subunits. These subunits are further linked via corners by [Sb_3_Cl_6_]^3–^ units forming the three-dimensional corner-sharing network (see Figure S4). The framework topology can be described with the point symbol {12.14^2^} 3{12} {14^3^}. This framework topology has not been reported before for metal halide frameworks, and it is the first framework with this topology according to the TOPOS database.^72^ Besides the framework composed of [Sb^III^Cl_6_]^3–^ units, the structure contains heavily disordered, isolated [Sb^V^Cl_6_]^−^ octahedra present in the structure. The octahedra are located along the 3-fold rotational axis of the structure, and the chlorides are disordered over 18 positions and refined to an occupancy of 0.33 resulting in a nominally distorted octahedral coordination. The disorder is still present if the structure is solved in a subgroup of *Pa*3̅. Therefore, *Pa*3̅ was chosen as the appropriate space group. The Sb–Cl bond lengths are in the range of 2.256(6)–2.475(6) Å, which indicates the oxidation state +5 for Sb.^13,58–60^ Since this compound contains antimony in distinct oxidation states and shows intervalence absorption in optical spectroscopy, we classify it as a Robin-Day Class II compound (see below).

**Figure 3:**
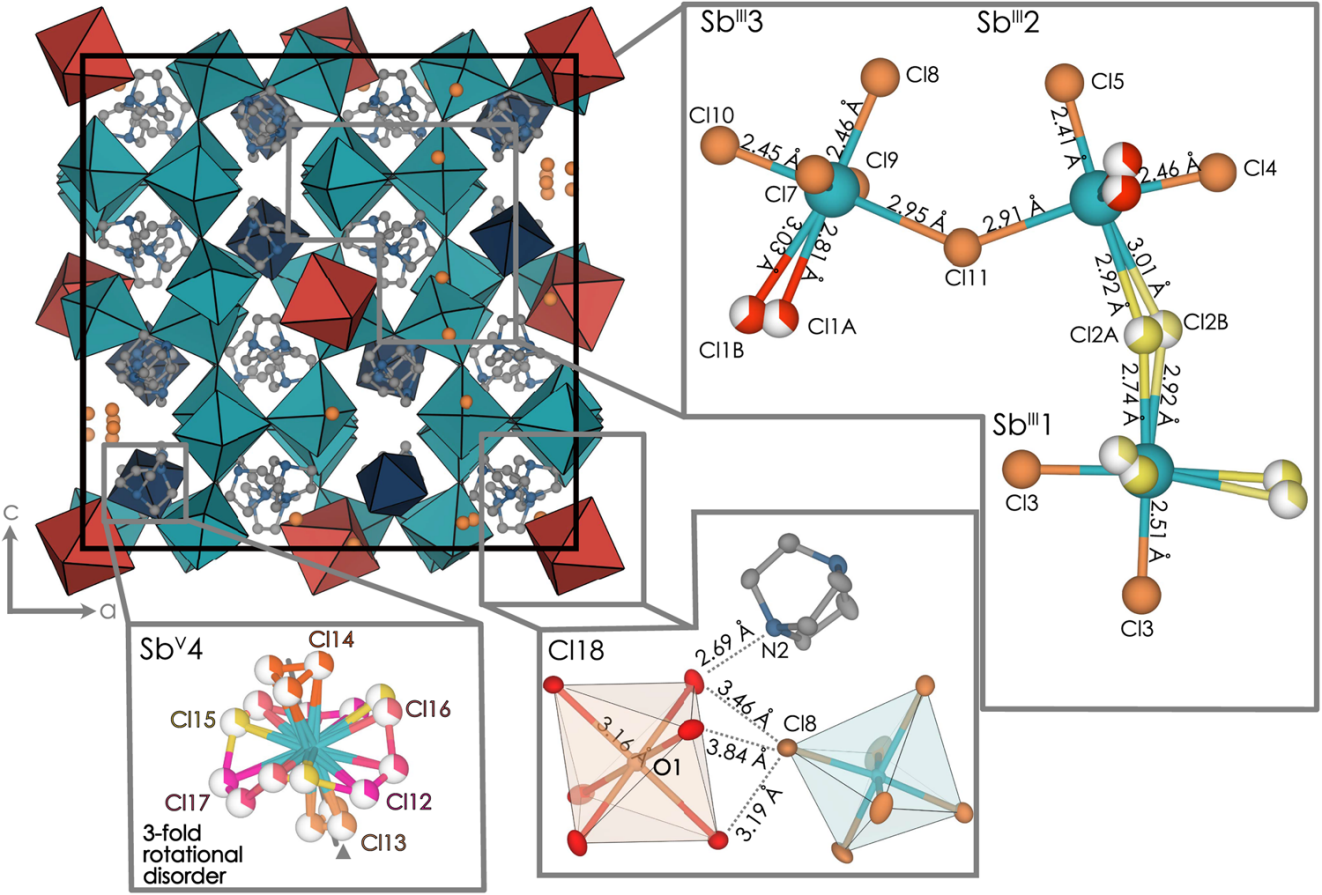
Crystal structure of compound **2** at 101 K with [Cl­(H_2_O)_6_]^−^ clusters in orange, and [Sb^III^Cl_6_]^3–^ units of the three-dimensional framework in mint. Idealized octahedra instead of the disordered [Sb^V^Cl_6_]^−^ units are shown in dark blue in the unit cell. Chlorine atoms are shown in orange, nitrogen atoms in light blue, carbon atoms in gray, and oxygen atoms in red. Positional disorder of chloride, disordered water molecules, disorder in one of the DABCOH_2_ cations, and hydrogen atoms are omitted for clarity. Anisotropic displacement ellipsoids are drawn at 50% probability level.

### Chloride Hydrate Cluster

Besides a framework structure, compound **2** features an internal chloride hydrate cluster [Cl­(H_2_O)_6_]^−^ with octahedral geometry (see Figure 3). The chloride is situated on a special position (Wyckoff 4b); accordingly, the cluster is highly symmetric with no distortion. So far only three discrete chloride hydrate clusters have been reported, namely [Cl­(H_2_O)]^−^,^20–22^ [(Cl)_2_(H_2_O)_4_]^2–^,^23^ and [(Cl)_2_(H_2_O)_6_]^2–^.^24^ The cluster reported here is the first internal monohalide hydrate cluster.^20,22,73^ The Cl–O distance of 3.159(5) Å at 101 K is in the same range as the three previously identified chloride hydrate clusters (2.936–3.253 Å).^20–24^ Interestingly, isolated internal chloride hydrate clusters have been predicted to be unstable and so far, no compound containing this unit has been reported.^25,26^ The chloride is always located on the surface of the cluster to minimize the number of chloride – water hydrogen bonds. Increasing the number of hydrogen bonds weakens each hydrogen bond, destabilizing the cluster.^25^ In compound **2**, the combination of inorganic framework and the DABCOnium cation seem to stabilize the otherwise unstable internal cluster. The O–N distance to the closest DABCOnium cation is 2.689(9) Å, while the O–Cl distances to the three-dimensional framework are 3.190(7) −3.842(8) Å (see Figure 3). The DABCOnium cation acts as the hydrogen bond donor toward the water molecules in the chloride-water cluster, thus indirectly adding to the stabilization of the cluster. The O–N distance of 2.689(9) Å is in the expected range for medium-strong hydrogen bonds and the interaction can be classified according to Jeffrey^74^ and Steiner^75^ as mostly electrostatic in nature. In the case of the water–chloride interactions with the chloride located in the inorganic framework, water acts as the hydrogen bond donor and chloride as the acceptor. Due to the distance, these interactions can be classified as weak, mostly electrostatic interactions.^74,75^ The geometrical constraints of the inorganic framework in combination with mostly electrostatic hydrogen bonding between the DABCOnium cations and water, as well as water and the chloride of the inorganic framework provide the conditions to stabilize the internal chloride-water cluster (see Table S3 for hydrogen bonding in compound **2**).

To further probe the structural properties of the chloride hydrate cluster, ssNMR and IR spectroscopy were performed. In the ^35,37^Cl MAS ssNMR spectrum one signal is visible (see Figure 4b). The MAS signal was fitted as a single site chlorine atom with a quadrupolar coupling constant *C*_Q_ = 1.97 MHz, asymmetry parameter η_Q_ = 0.21 and an isotropic shift δ_iso_ = 84.24 ppm. The static sample signal was analyzed with the *C*_Q_, η_Q_, and δ_iso_ fixed at the values determined from the MAS sample. This yielded a chemical shift tensor span of Ω = 13.42 ppm and a skew of κ = 0.74. The isotropic chemical shift is in the expected range of hydrochloride salts of amines and alkaline earth chloride hydrates, and therefore we assign this signal to the chloride hydrate cluster.^76,77^ To the best of our knowledge, no chemical shifts have been reported for chloride hydrate clusters, and hence this is the first NMR characterization of such a cluster. Therefore, a direct comparison with the other reported chloride hydrate clusters is not possible. To corroborate the results, the chemical shifts were quantum chemically calculated, yielding 80.73 ppm. This is in good agreement with the experimental value of 84.24 ppm. The signals of the chlorides in the three-dimensional framework are not visible due to the lower site symmetry (Wyckoff position Sb1, Sb4:8c, Sb2, Sb3:24d), resulting in substantially larger quadrupolar coupling constants. Indeed, the calculated quadrupolar coupling constants for the chlorides in the three-dimensional framework are five to nine times larger than that of the chloride in the hydrate cluster (see Table S9). Such large couplings lead to severe second-order quadrupolar broadening, potentially spanning thousands of ppm, as demonstrated by Sarkar et al. for metal halide perovskites and related materials.^78^ Such a large broadening of the spectra could be the cause for the absence of the signals of the three-dimensional framework and the isolated [Sb^V^Cl_6_]^−^ unit in the ^35, 37^Cl ssNMR MAS spectrum.

**Figure 4:**
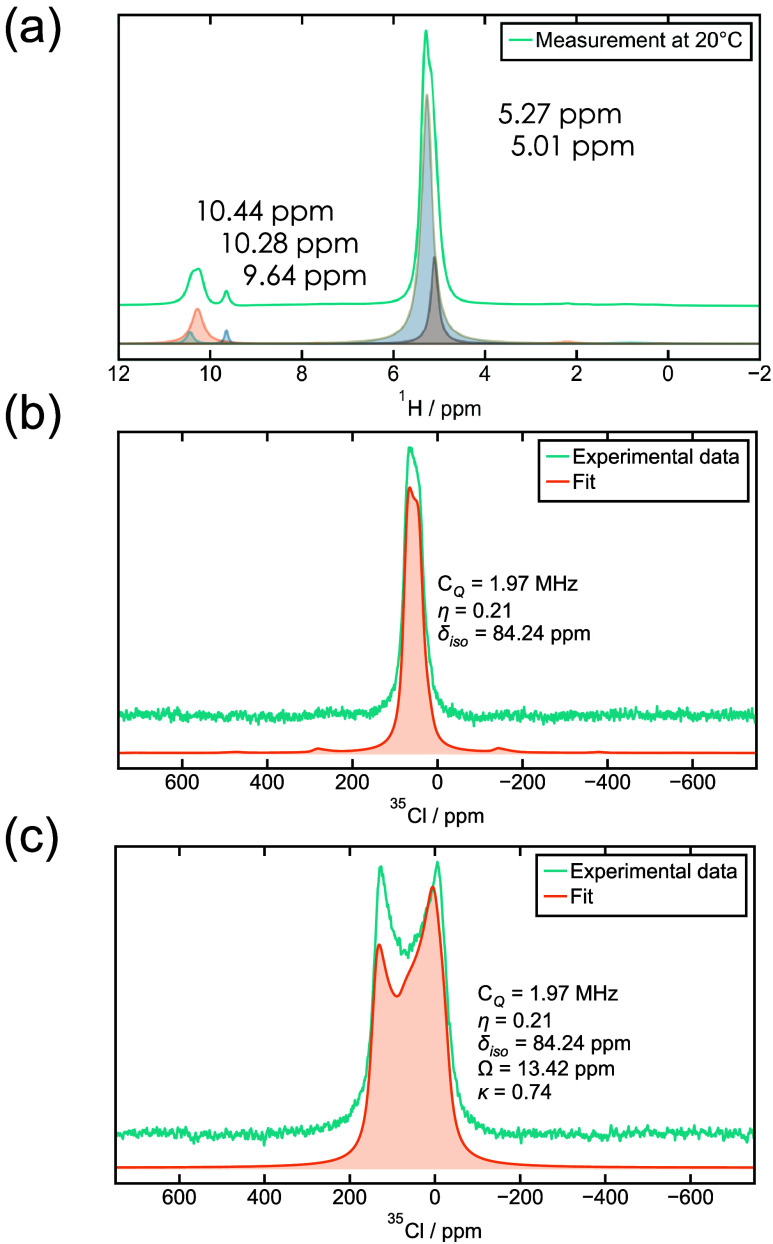
ssNMR spectra of compound **2**. (a) ^1^H MAS NMR spectrum with five signals fitted at 10.42, 10.24, 9.65, 5.30, and 5.12 ppm. Acquisition parameters: rotation speed 55 kHz, *T* = 20 °C. (b) ^35^Cl MAS NMR spectrum fitted as a single site chlorine. (c) ^35^Cl static Hahn echo of compound **2**.

In the ^1^H ssNMR MAS spectrum, the signals of the chloride hydrate cluster, hydronium ions, the disordered crystal water and organic cations are visible (see Figure 4a). Due to the CH and NH correlation spectra (see Figures S18 and S19), we assign the signals at 5.30 and 5.12 ppm to the CH_2_ groups of the DABCOnium cation and the signals at 9.65, 10.24, and 10.42 ppm to the NH groups. In the crystal structure there are two different DABCOnium cations with four crystallographically unique N. In the ^1^H–^15^N HSQC spectrum four NH environments are visible, while in the CP ^1^H–^15^N 2D spectrum three environments are visible, which suggests that one NH group is rather mobile (see Figure S18). One of the NH groups is located close to the chloride cluster and has mostly electrostatic interactions with the hydrate shell of the cluster. Due to this proximity the NH proton could be mobile and undergo proton exchange with the water nearby and explain the absence of a fourth signal in the CP ^1^H–^15^N 2D spectrum. In the temperature-dependent ^1^H NMR (see Figure S20), it can be seen that upon cooling the sample, signals at around 12.1 ppm as well as around 4 ppm arise. In the literature, isolated water molecules are reported at 2.2 ppm while signals at 5.6 ppm can be assigned to structural water, and signals around 11 ppm can be assigned to H_3_O^+^.^79–82^ We tend to assign the signals at 4 and 12.1 ppm to the water of chloride hydrate cluster and hydronium in the structure as they do not show any coupling to carbon or nitrogen (see Figures S17 and S18).^80,83^ The signal at 4 ppm shows coupling with the signal at 10.4 ppm (see Figures S18 and S19). The latter signal is assigned to a NH group and only the water of the chloride hydrate cluster is in proximity to an NH group; hence we assign the signal at 4 ppm to the water in the chloride hydrate cluster.^82^ Due to these assignments, we attribute the signal at 5.12 ppm to CH_2_ groups on the same DABCOnium in proximity to the chloride hydrate cluster (see Figure S17). The IR spectrum shows several peaks which can be assigned to NH, CH_2_, and OH vibrations (see Figure S33). The strong signal at 3022 cm^–1^ can be assigned to CH_2_ stretching modes of the organic cation.^27^ The broad signal around 3415 cm^–1^ can be assigned to O–H stretching vibrations in the chloride hydrate cluster as well as H_3_O^+^,^84^ in good agreement with the literature, as well as to the N–H stretching modes of the organic cation.^25,27^ The signal at 1620 cm^–1^ can be assigned to the O–H bending mode of water.^27,85^

Thermogravimetric analysis was performed to assess the thermal stability of compound **2**. These measurements revealed thermal behavior expected for a hydrate, with gradual loss of water with increasing temperature and subsequent decomposition (see Figures S34 and S35). Upon heating to 400 °C, the sample loses 74 wt % and decomposes. In a first step up to 175 °C, the sample shows a mass loss of 3 wt %, which could be associated with the loss of crystal water as well as some water from the chloride-water cluster. The theoretical weight loss for all water would be 9.5 wt %. Thus, in this step only parts of the water are lost. In a second step up to 280 °C, the sample loses further 13 wt %, which could be attributed to the removal of further water, DABCOH_2_ and SbCl_3_. Above 280 °C the sample decomposes.

To further corroborate our inferences about the chloride hydrate cluster, we investigated the closely related compound (DABCOH_2_)_4_Sb_2_^III^Cu_2_^II^Cl_18_(H_2_O)_4_ for comparison. The compound is obtained for the highest reactant concentration and has been recently described in the literature.^28^ The structure was described as a layered structure consisting of an inorganic layer built by [Cu^II^Cl_6_]^4–^ units and [Sb^III^Cl_6_]^3–^ and a second inorganic layer with [Sb^III^Cl_5_]^2–^, water and chloride ions. These layers are separated by the organic cation. Upon closer inspection of its structure, we find that the compound contains one isolated chloride ion, which is coordinated by water molecules (see Figure S8). The Cl9–O distance is 3.230(6) Å, which is again in the range of that observed for chloride hydrate clusters. In contrast to compound **2**, however, the coordination is tetrahedral. The coordinating water molecules are also in proximity to the [Sb^III^Cl_5_]^2–^ units with SbCl–O distances of 3.261(6) Å, which again is in the range observed for chloride hydrate clusters. Therefore, rather than describing the second inorganic layer based on isolated [Sb^III^Cl_5_]^2–^ units, isolated water molecules, and isolated chloride ions, it should be described as a layer built from alternating, hydrogen-bonded [Sb^III^Cl_5_]^2–^ and [Cl­(H_2_O)_4_]^−^ units. The tetrahedral [Cl­(H_2_O)_4_]^−^ units are connected in a corner-sharing fashion to the square-pyramidal [Sb^III^Cl_5_]^2–^ units. The solid-state MAS and static ^35^Cl NMR spectra of (DABCOH_2_)_4_Sb_2_^III^Cu_2_^II^Cl_18_(H_2_O)_4_ can again be fitted with one single signal, in agreement with our observations for compound **2** (see Figure 5a,b). The isotropic chemical shift δ_iso_ = 74.73 ppm is in the same range as for compound **2** and thus can be ascribed to [Cl­(H_2_O)_4_]^−^.

**Figure 5:**
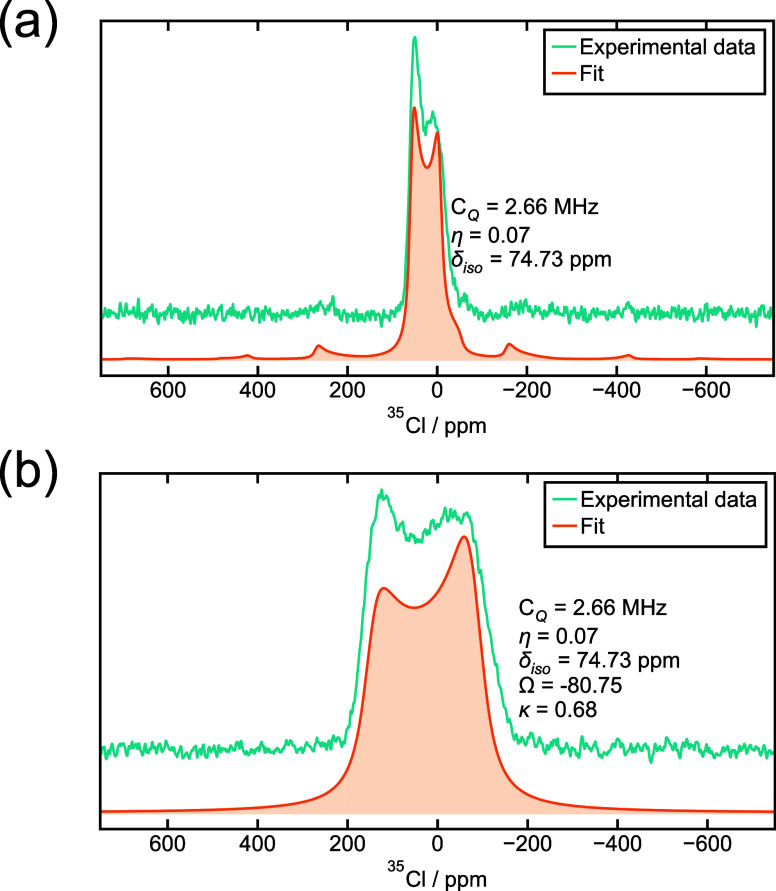
ssNMR spectra of (DABCOH_2_)_4_Sb_2_^III^Cu_2_^II^Cl_18_(H_2_O)_4_. (a) ^35^Cl MAS NMR fitted as a single site chlorine atom. (b) ^35^Cl static Hahn echo.

The optical properties of the three compounds are dominated by their inorganic substructure due to the absence of a π-system in the organic cation. The Kubelka–Munk transformed diffuse reflectance spectra of **1** and **2** are shown in Figure 6.

**Figure 6:**
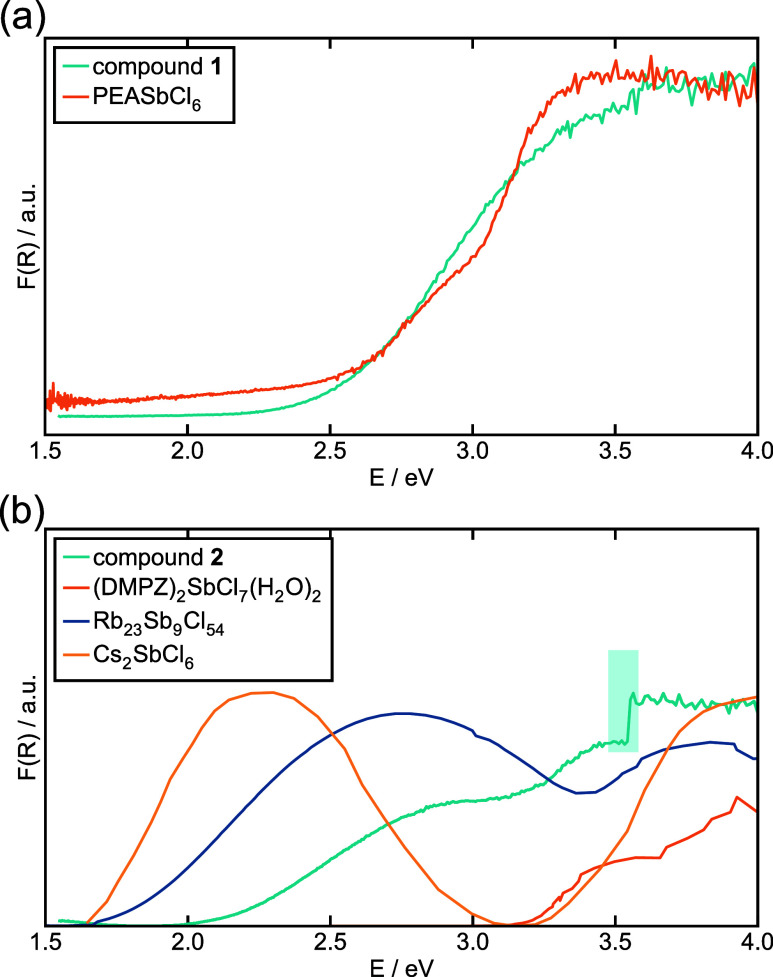
(a) Kubelka–Munk transformed diffuse reflectance spectra of compound **1**, and PEASb^V^Cl_6_.^13^ (b) **2**, (DMPZ)_2_Sb^III^Cl_12_(H_2_O)_2_ ^63^ Rb_23_Sb_7_^III^Sb_2_^V^Cl_54_,^16^ Cs_2_Sb^III/V^Cl_6_,^18^ normalized between minimum and maximum absorption. The signal marked by a light mint area is due to the lamp changing during the measurement.

The absorption of the yellow compound **1** is in the blue range of the spectrum, similar to compounds with isolated [Sb^V^Cl_6_]^−^ units reported in the literature, like PEASb^V^Cl_6_, which is also shown in Figure 6a for comparison.^13,86^ Unfortunately, for most of the reported Sb^V^ compounds optical characterization is absent, but white to yellow has been qualitatively reported as their color. The optical band gap of compound **1** was determined from a Tauc plot assuming a direct allowed transition, yielding *E*_g_ = 2.78 eV, consistent with its yellow color and the absence of low-energy absorption features (see Figure S30). In the orange compound **2**, the absorption in the blue and UV range is similar to **1**, but there is additional absorption in the lower-energy regime compared to compound **1**, which we assign to the intervalence absorption of the material (see Figure 6b). The high-energy absorption is similar, for example, to the absorption in (DMPZ)_2_Sb^III^Cl_12_(H_2_O)_2_ ^63^ (DMPZ = *N*,*N*’-dimethylpiperazine), which contains isolated [Sb^III^Cl_6_]^3–^ units. In spite of differences in the inorganic substructures of these two compounds, we assign the high-energy feature to the absorption of the inorganic framework. The low-energy absorption is likely due to the intervalence charge transfer. The intervalence absorption is in the same range as that observed for the mixed-valence antimony compound Rb_23_Sb_7_^III^Sb_2_^V^Cl_54_, but not as intense. This may be due to the three-dimensional framework of [Sb^III^Cl_6_]^3–^ units in compound **2**, compared to the more common isolated units reported in Rb_23_Sb_7_^III^Sb_2_^V^Cl_54_.^10,13,16^ The distances between the Sb^III^ and Sb^V^ sites in compound **2**, Cs_2_Sb^III/V^Cl_6_, and Rb_23_Sb_7_^III^Sb_2_^V^Cl_54_ are shown in Table S4. Despite compound **2** having the shortest distance between Sb^III^ and Sb^V^ sites its intervalence charge transfer band is the weakest. This suggests that the ratio of Sb^III^ to Sb^V^ is responsible for the strength of the intervalence charge transfer. In compound **2** Sb^III^:Sb^V^ is 14:2 while in Cs_2_Sb^III/V^C_l6_ the ratio is 1:1 and in Rb_23_Sb_7_^III^Sb_2_^V^Cl_54_ 7:2. There are fewer intervalence charge transfer active pairs in compound **2**, which results in a weaker intervalence charge transfer. For compound **2** we also assumed a direct allowed transition, but a straightforward Tauc analysis is complicated by the presence of two overlapping absorption features. The high-energy onset, attributed to the three-dimensional framework yields an absorption onset of 2.36 eV (see Figure S31). The lower-energy feature is assigned to the intervalence charge transfer, consistent with the Robin-Day Class II classification of compound **2**.

## Conclusions

New HOI antimony halides have been prepared by precipitation from hydrochloric acid in the presence of Cu^II^Cl_2_ and oxygen. Increasing the concentration of the starting materials has a distinct influence on the nature of the inorganic building units, their oxidation states, and the dimensionality of the inorganic substructure. While compound **1** contains [Sb^V^Cl_6_]^−^ units separated by the organic DABCO cation, compound **2** exhibits an unusual structure with a three-dimensional framework of [Sb^III^Cl_6_]^3–^ units, along with disordered [Sb^V^Cl_6_]^−^ units and an unexpected chloride hydrate cluster. Both the material and the cluster were characterized using single-crystal XRD, ssNMR, IR spectroscopy, and corroborated by quantum chemical NMR calculations. Compound **2** is a HOI antimony chloride compound featuring a three-dimensional framework with a new network topology. The octahedral chloride hydrate cluster [Cl­(H_2_O)_6_]^−^ is also the first example of an internal halide hydrate cluster with the halide being located in the center, rather than the surface of the cluster. The cluster appears to be stabilized by weak interactions with the surrounding framework and DABCOnium cations. NMR characterization of (DABCOH_2_)_4_Sb_2_^III^Cu_2_^II^Cl_18_(H_2_O)_4_ reported by Liu et al.^28^ revealed another chloride hydrate cluster, which is connected to [Sb^III^Cl_5_]^2–^, resulting in a layered motif. Comparison of the optical spectra of the compounds shows that the mixed valency of the inorganic substructure leads to a pronounced red-shift due to intervalence charge-transfer transitions. Our study reveals a rich phase space in HOI antimony halides that can be accessed through compositional control.

## Supplementary Material



## Data Availability

Raw data of PXRD, ssNMR, FT-IR, STA, UV–vis, scripts for the figure generation, input files for Pawley refinements, and quantum chemical calculation results can be found in https://gitlab.gwdg.de/jakob.blahusch/antimony_halide_water_cluster. The authors have cited additional references within the Supporting Information.^87^

## Notes

Experimental information, crystallographic data, ssNMR, FT-IR, and STA (PDF)

## §

Institute of Science and Technology Austria, Am Campus 1, A-3400 Klosterneuburg, Austria

## ∥

PSI Center for Neutron and Muon Sciences, Paul Scherrer Institute, PSI, 5232 Villigen, Switzerland.
