# Supplementary material for: Tuning Connectivity in Hybrid Organic–Inorganic Antimony Halides through Reactant Concentration Effects
Source: Inorg Chem. 2026 Jul 11;65(29):16804–14. doi: 10.1021/acs.inorgchem.6c01714 (PMC13418105; doi:10.1021/acs.inorgchem.6c01714)
Supplement: Supplementary file 1 [file ic6c01714_si_001.pdf]

Supporting Information for: Tuning Connectivity in Hybrid Organic-Inorganic Antimony Halides through Reactant Concentration Effects

Jakob Blahusch[1,2], Julia Rauh[1,2], Petra Rovo[2,3], Igor Moudrakovski[1], Douglas H. Fabini[1,4], Daniel Graf[2], Christian Ochsenfeld[2], and Bettina V. Lotsch*[1,2]

[1]Max Planck Institute for Solid State Research, Heisenbergstraße 1, 70569 Stuttgart, Germany
[2]Department of Chemistry, Ludwig-Maximilians-Universität München, Butenandtstraße 5–13, 81377 München, Germany
[3]Present Address: Institute of Science and Technology Austria, Am Campus 1, A-3400 Klosterneuburg, Austria
[4]Present Address: PSI Center for Neutron and Muon Sciences, Paul Scherrer Institute, 5232 Villigen PSI, Switzerland

*Email: b.lotsch@fkf.mpg.de

S1 Experimental

Table S1: Used chemicals with supplier and purity.

Chemical	Supplier	Purity
Sb_2O_3	Acros Organics	$\geq$ 99 %
DABCO	Merck	$\geq$ 98 %
anhydrous $CuCl_2$	Fluka	$\geq$ 97 %
conc. HCl	Merck	$\geq$ 95 %

S1.1 Sb_2O_4

Sb_2O_4 was prepared by heating Sb_2O_3 in air to 550 °C for 12 hours. The PXRD pattern of the obtained colorless powder is in good agreement with the literature (see Figure S1).[1]

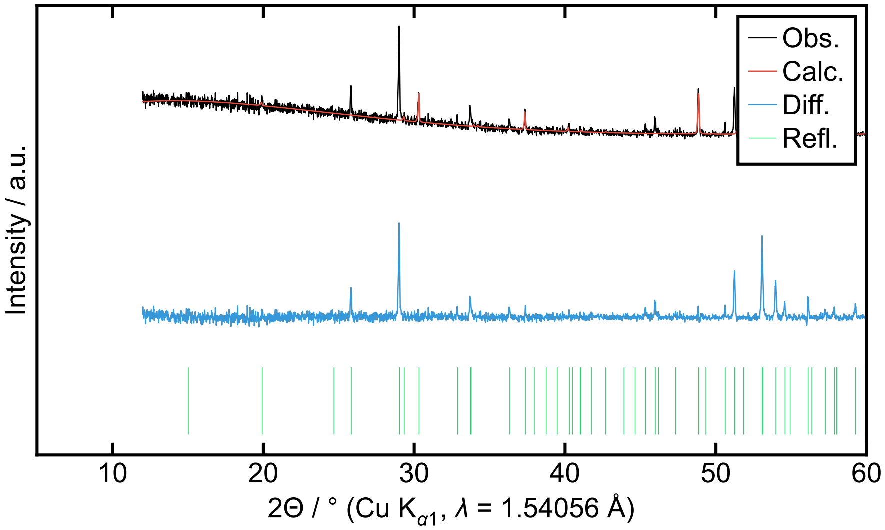

Figure S1: Pawley fit of the prepared Sb_2O_4 using the crystal structure of Sb_2O_4.[1] R_{wp} = 22.03 , GoF = 1.45, refined lattice parameters: a = 12.11 Å, b = 4.81 Å, c = 5.38 Å, β = 103.85°.

S1.2 Concentrations of the reactants and volume of conc. HCl

Table S2: Concentrations of the reactants and volume of conc. HCl used for the synthesis of compound **1**, **2**, and $(DABCOH_2)_4Sb^{III}{}_2Cu^{II}{}_2Cl_{18}(H_2O)_4$.

	compound **1**	compound **2**	$(DABCOH_2)_4Sb^{III}{}_2Cu^{II}{}_2Cl_{18}(H_2O)_4$
conc. DABCO/ mmol/mL	0.1	0.2	0.1
conc. Sb_2O_3/ mmol/mL	0.0125	0.025	0.05
conc. $CuCl_2$/ mmol/ mL	0.0125	0.025	0.1
Volume conc. HCl / mL	10	10	10

S2 Crystallographic Information

S2.1 Refinement details for 1

Compound **1** features positional disorder of two chlorides. Each of the chloride sites was refined as a split position with an occupancy of $\frac{1}{2}$.

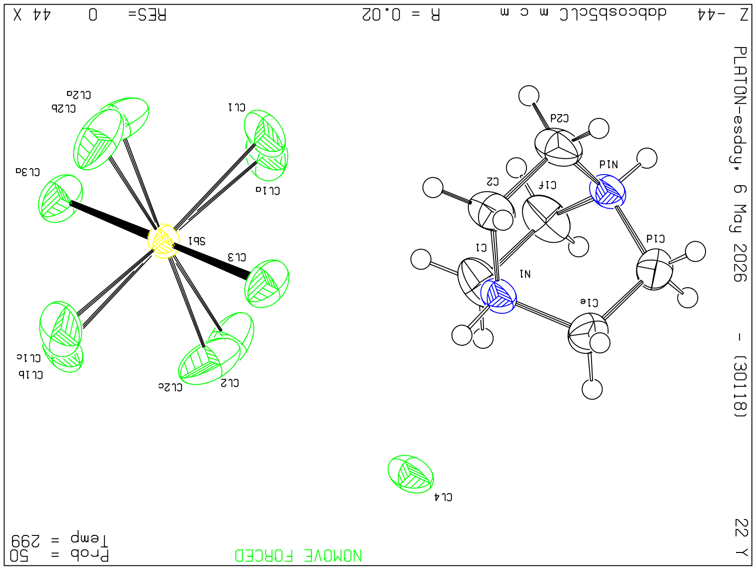

Figure S2: ORTEP (Oak Ridge Thermal-Ellipsoid Plot) drawing of compound **1**. Displacement ellipsoids are drawn at the 50% probability level.

S2.2 Refinement details for 2

Compound **2** features several sources of disorder. The structure contains one Sb^{V} (Sb4) site. Sb4 is modelled at three positions related by the local threefold symmetry axis, and its six coordinating Cl atoms are disordered over 18 positions due to the local threefold rotational symmetry of the $[Sb^{V}Cl_6]^-$ octahedron about the crystallographic threefold axis. The occupancy of each Cl position was fixed to $\frac{1}{3}$. The disordered Sb4 site and its associated Cl atoms were treated using `PART -1` in SHELXL to exclude them from bond angle and connectivity calculations. Cl1 and Cl2 sites were refined with a split position. One $DABCOH_2{}^{2+}$ cation shows evidence of rotational disorder about the molecular N–N axis. A two-component disorder model was attempted but could not be refined satisfactorily and was therefore abandoned; the carbon atoms of the affected cation show enlarged anisotropic displacement parameters as a consequence. The oxygen atom of the crystallographically located water molecule (O1) was refined freely, with O–H distances and the H–O–H angle restrained using `DFIX` (target d(O–H) = 0.96 Å) and `DANG` (target d(H···H) = 1.54 Å) instructions. The remaining disordered solvent molecules were treated using the OLEX2 solvent mask, which identified 1220 electrons in a volume of 1564 Å^3 in 2 crystallographically independent void types per unit cell, consistent with the presence of 0.9 H_2O and 4.1 H_2O per asymmetric unit, accounting for approximately 1200 electrons per unit cell. The hydronium ions (H_3O^+) included in the molecular formula were not located crystallographically but were added for charge balance.

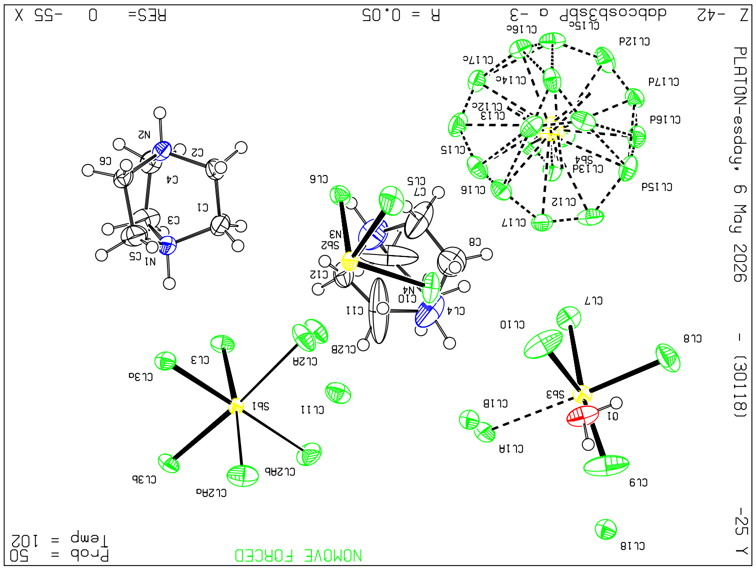

Figure S3: ORTEP drawing of compound **2**. Displacement ellipsoids are drawn at the 50% probability level.

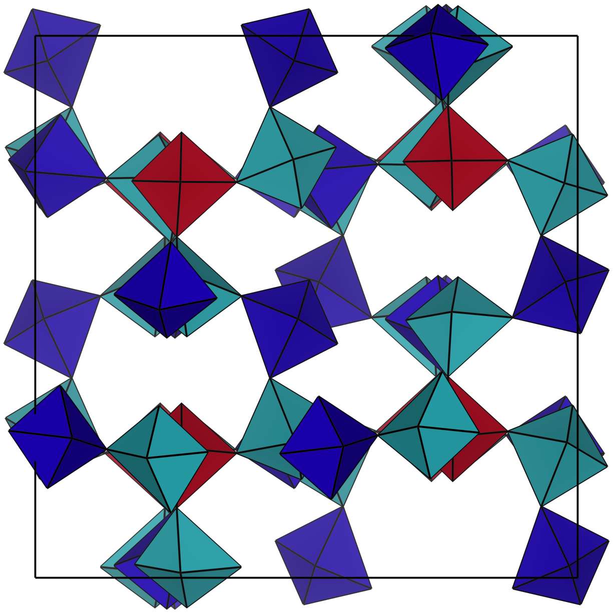

Figure S4: Three-dimensional framework of $[Sb^{III}Cl_6]^{3-}$ units in compound **2**. $[Sb_1Cl_6]^{3-}$ units are shown in red, $[Sb_2Cl_6]^{3-}$ units in mint, and $[Sb_3Cl_6]^{3-}$ units in blue.

Figure S5 to S7 show the single-crystal X-ray diffraction precession images of compound **2**.

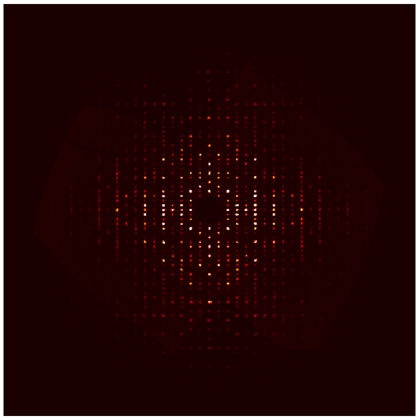

Figure S5: Calculated precession image from compound **2** SXRD data corresponding to the 0kl plane.

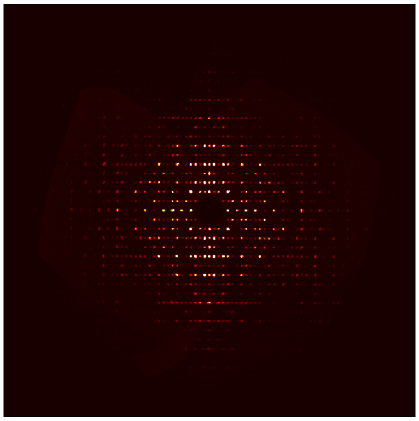

Figure S6: Calculated precession image from compound **2** SXRD data corresponding to the h0l plane.

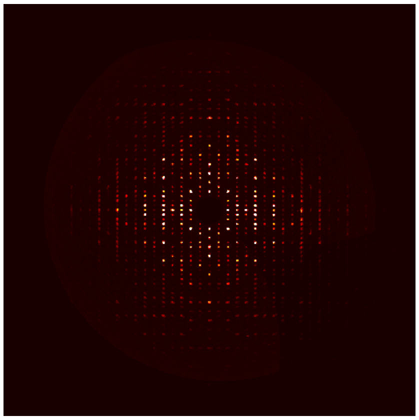

Figure S7: Calculated precession image from compound **2** SXRD data corresponding to the hk0 plane.

S2.3 Hydrogen bonding in compound 2

Table S3: Hydrogen bonds in compound **2**.

D	H	A	d(D–H) /Å	d(H···A) /Å	d(D···A) /Å	∠D–H···A /°
O–H··· A and N–H··· A						
O1	H1C	Cl18	0.970(10)	2.27(5)	3.159(5)	151(8)
N1	H1	Cl14[a,1]	1.00	2.90	3.544(7)	122.7
N1	H1	Cl14[a,2]	1.00	2.94	3.565(6)	121.4
N2	H2	O1[2]	1.00	1.80	2.693(7)	146.7
N3	H3	Cl7	1.00	3.04	3.646(9)	119.8
N4	H4	Cl1B	1.00	2.06	3.024(13)	161.6
C–H··· A						
C1	H1B	Cl11[3]	0.99	3.05	3.770(8)	130.8
C2	H2A	Cl11[3]	0.99	2.88	3.700(7)	141.1
C3	H3A	Cl12[a,2]	0.99	2.56	3.480(10)	154.4
C3	H3A	Cl15[a,1]	0.99	2.67	3.356(9)	126.3
C3	H3A	Cl17[a,2]	0.99	2.79	3.773(10)	173.2
C5	H5B	Cl14[a,2]	0.99	3.03	3.724(10)	128.6
C5	H5B	Cl16[a,2]	0.99	2.85	3.755(10)	152.9
C7	H7A	Cl13[a]	0.99	2.67	3.509(15)	143.0
C7	H7A	Cl13[a,4]	0.99	3.02	3.734(14)	129.6
C8	H8B	Cl13[a]	0.99	3.07	3.714(12)	123.8
C8	H8B	Cl13[a,4]	0.99	2.81	3.604(12)	137.8
C10	H10A	Cl11	0.99	2.78	3.636(9)	145.2
C12	H12A	Cl2B	0.99	2.82	3.35(3)	114.7

[1] $1-z,\ 1-x,\ 1-y$
[2] $\frac{3}{2}-y,\ \frac{1}{2}+z,\ +x$
[3] $\frac{1}{2}+z,\ \frac{3}{2}-x,\ 1-y$
[4] $1-y,\ \frac{1}{2}+z,\ \frac{1}{2}-x$
[a] Disordered Sb4 cluster (occupancy $\frac{1}{3}$ each)

S2.4 Antimony distances in 2 and related compounds

Table S4: Shortest Sb^{III}–Sb^{V} distances in compound **2**, $Cs_2Sb^{III/V}Cl_6$,[2] and $Rb_{23}Sb^{III}{}_7Sb^{V}{}_2Cl_{54}$.[3]

Compound	$d(Sb^{III}–Sb^{V})$ /Å
Compound **2**	6.76 Å
$Cs_2Sb^{III/V}Cl_6$	7.60 Å
$Rb_{23}Sb^{III}{}_7Sb^{V}{}_2Cl_{54}$	7.43 Å

S2.5 $(DABCOH_2)_4Sb^{III}{}_2Cu^{II}{}_2Cl_{18}(H_2O)_4$

The bond lengths of $[Cl(H_2O)_4]^-$ units in $(DABCOH_2)_4Sb^{III}{}_2Cu^{II}{}_2Cl_{18}(H_2O)_4$ are shown in Figure S8.

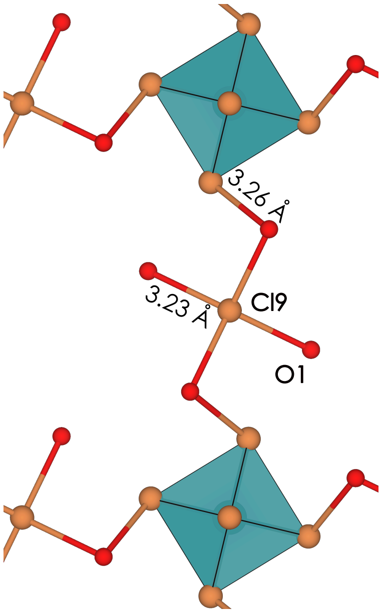

Figure S8: $[Cl(H_2O)_4]^-$ units and square pyramidal $[Sb^{III}Cl_5]^{2-}$ units (shown in mint) in $(DABCOH_2)_4Sb^{III}{}_2Cu^{II}{}_2Cl_{18}(H_2O)_4$.[4] Oxygen atoms are shown in red and chlorine atoms in orange.

S3 Powder X-Ray diffraction

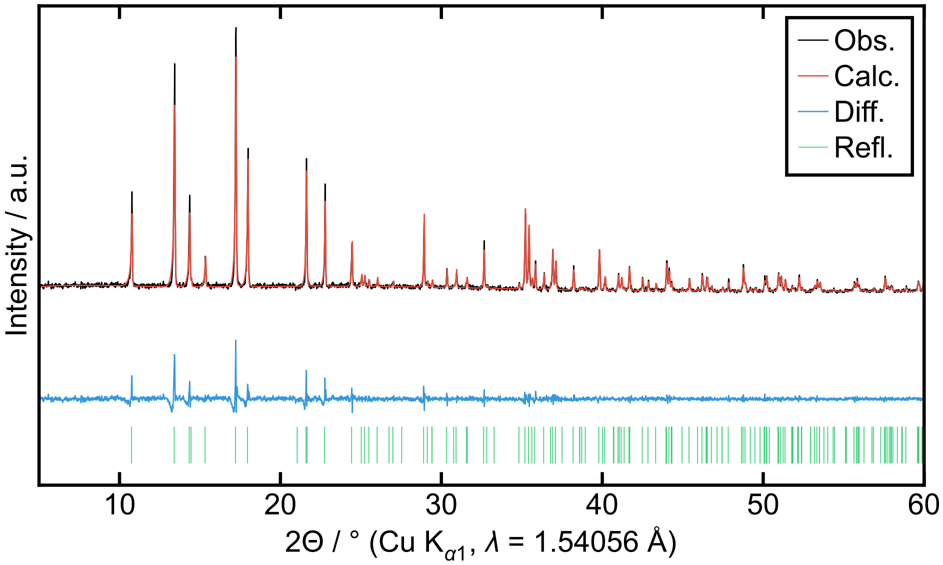

Figure S9: Pawley fit for compound **1**. R_{wp} = 15.86 , GoF = 1.03, refined lattice parameters: a = 7.81 Å, b = 12.34 Å, c = 16.45 Å.

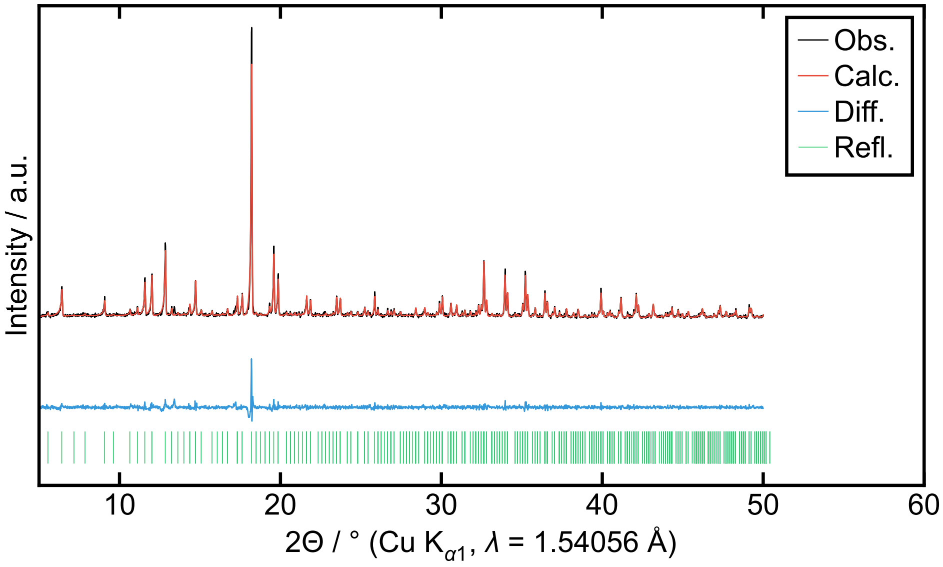

Figure S10: Pawley fit for compound **2**. R_{wp} = 23.27 , GoF = 1.69, refined lattice parameters: a = 27.56 Å.

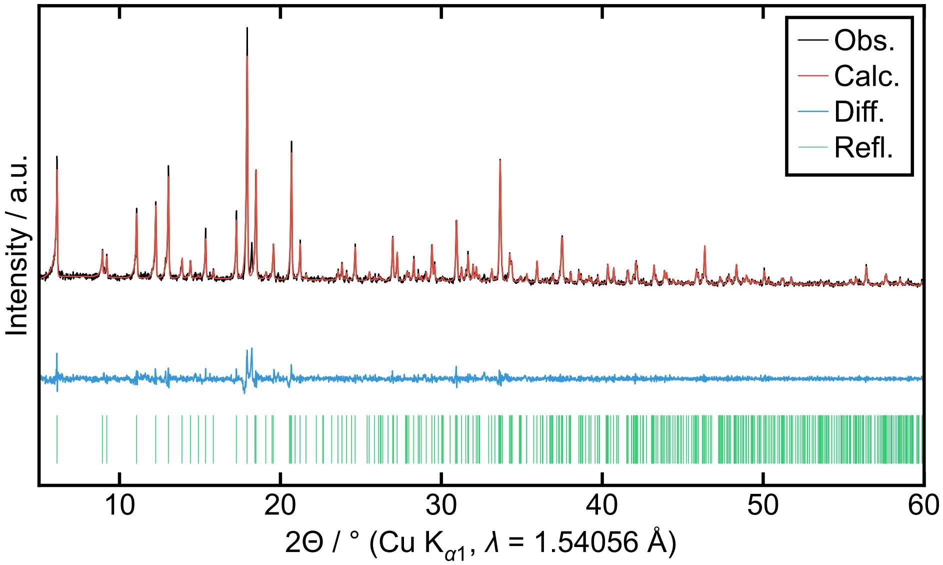

Figure S11: Pawley fit of product after synthesis in degassed solvent using the crystal structure of $(DABCOH_2)_4Sb^{III}{}_2Cu^{II}{}_2Cl_{18}(H_2O)_4$.[4] $R_{wp} = 12.23$, GoF = 1.05, refined lattice parameters: $a = 13.53$ Å, $c = 28.89$ Å.

S4 Solid state NMR

S4.1 MAS NMR of compound 1

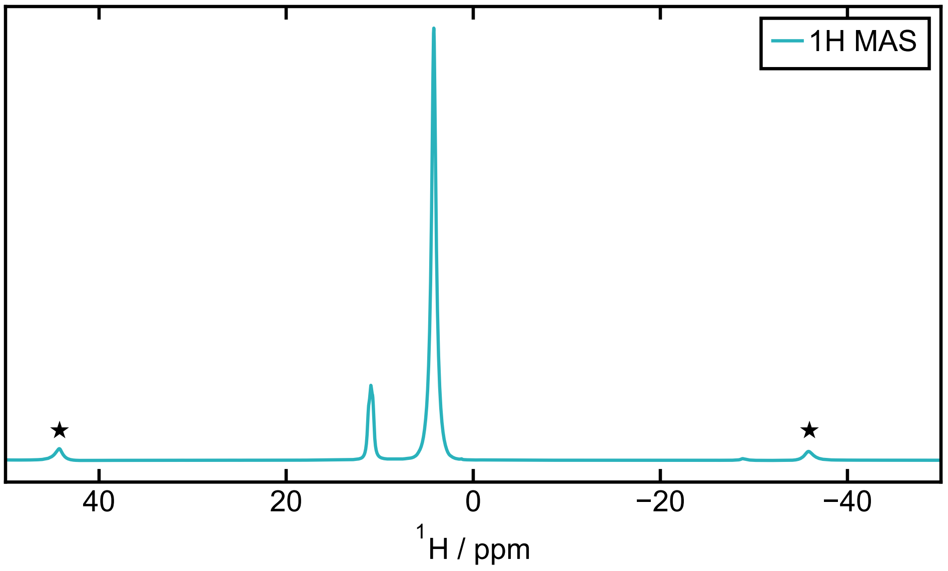

Figure S12: ^{1}H MAS NMR of compound **1** at 24 kHz MAS frequency. Spinning sidebands are labeled with a star.

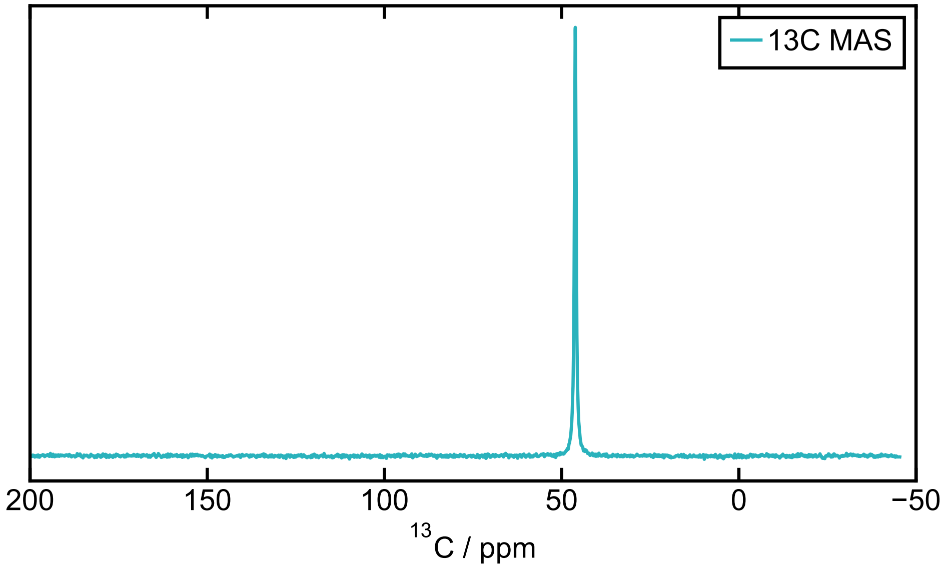

Figure S13: ^{1}H-^{13}C CP MAS of compound **1** at 15 kHz MAS frequency.

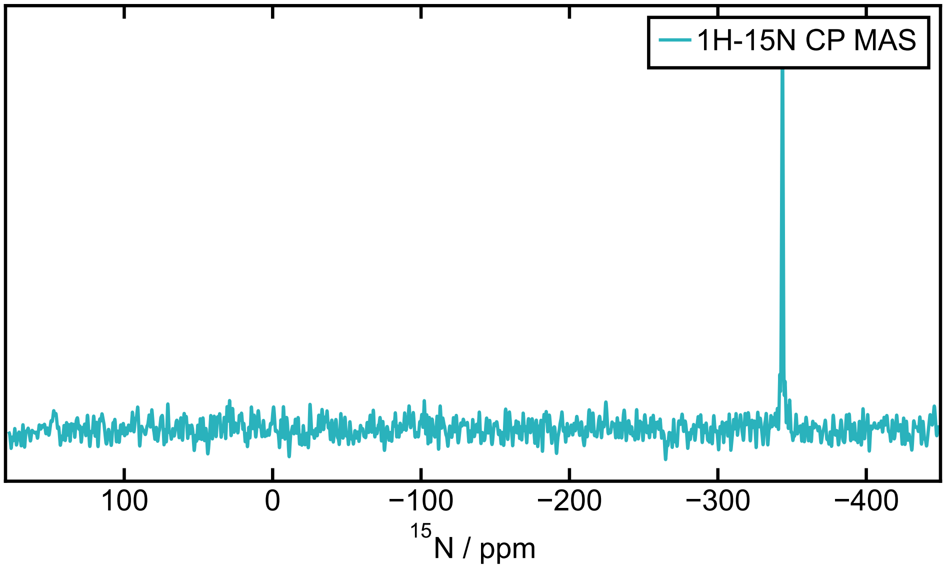

Figure S14: ^{1}H-^{15}N CP MAS of compound **1** at 15 kHz MAS frequency.

S4.2 Solid state NMR of compound 2

Fitting parameters for ^{35}Cl MAS ssNMR and ^{35}Cl static ssNMR

Table S5 and S6 contain the fitting parameters. The static signal was analysed by keeping C_Q, η and δ_{iso} fixed as determined from the MAS sample.

Table S5: Fitting parameters for ^{35}Cl MAS ssNMR of compound **2** obtained with ssNAKE.[5] Fit: Quadrupole, Units: ppm, I=3/2, MAS=Finite MAS, Satellites=False, Cheng=15, Angle=$arctan(\sqrt{2})$, Sidebands=32

δ_{iso}/ *ppm*	C_q/ *MHz*	η	Lorentz *kHz*	Gauss	LorentzST
84.24	1.97	0.21	1.1	0.000	1.00

Table S6: Fitting parameters for ^{35}Cl static ssNMR of compound **2** obtained with ssNAKE.[5] Fit: Quadrupole+CSA, Units: ppm, I=3/2, MAS=Static, Satellites=False, Cheng=15, Angle=$arctan(\sqrt{2})$, Sidebands=32

C_q/ *MHz*	η	δ_{iso}/ *ppm*	Ω/ ppm	κ	Lorentz/ *kHz*	Gauss
1.97	0.21	84.24	13.42	0.74	1.6	0.00

Fitting parameters for ^{37}Cl MAS ssNMR and ^{37}Cl static ssNMR

The static signal was analysed by keeping C_Q, η and δ_{iso} fixed as determined from the MAS sample.

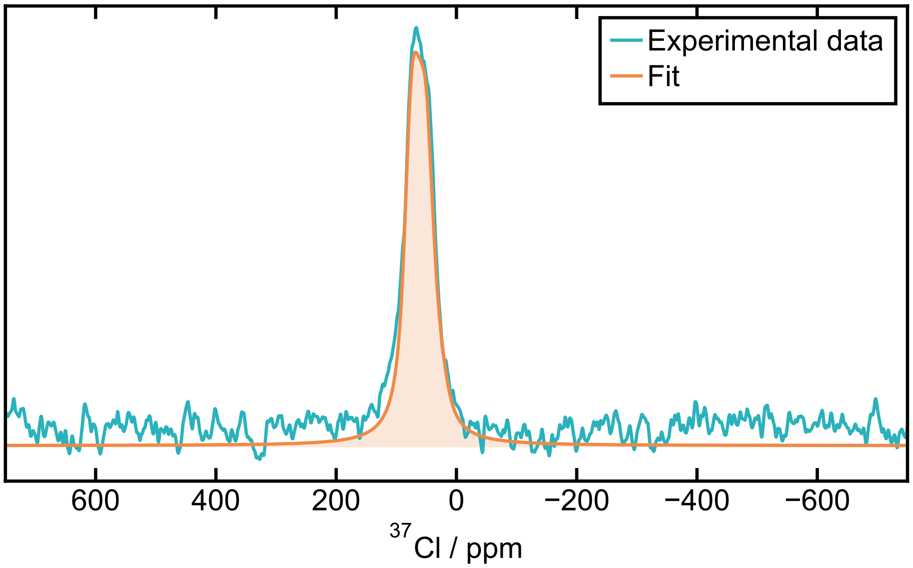

Figure S15: ^{37}Cl MAS ssNMR of compound **2** fitted as a single site chlorine atom.

Table S7: Fitting parameters for ^{37}Cl MAS ssNMR of compound **2** obtained with ssNAKE.[5] Fit: Quadrupole, Units: ppm, I=3/2, MAS=Finite MAS, Satellites=False, Cheng=15, Angle=$arctan(\sqrt{2})$, Sidebands=32

δ_{iso}/ *ppm*	C_q/ *MHz*	η	Lorentz *kHz*	Gauss	LorentzST
90.21	1.66	0.25	1.1	0.000	1.00

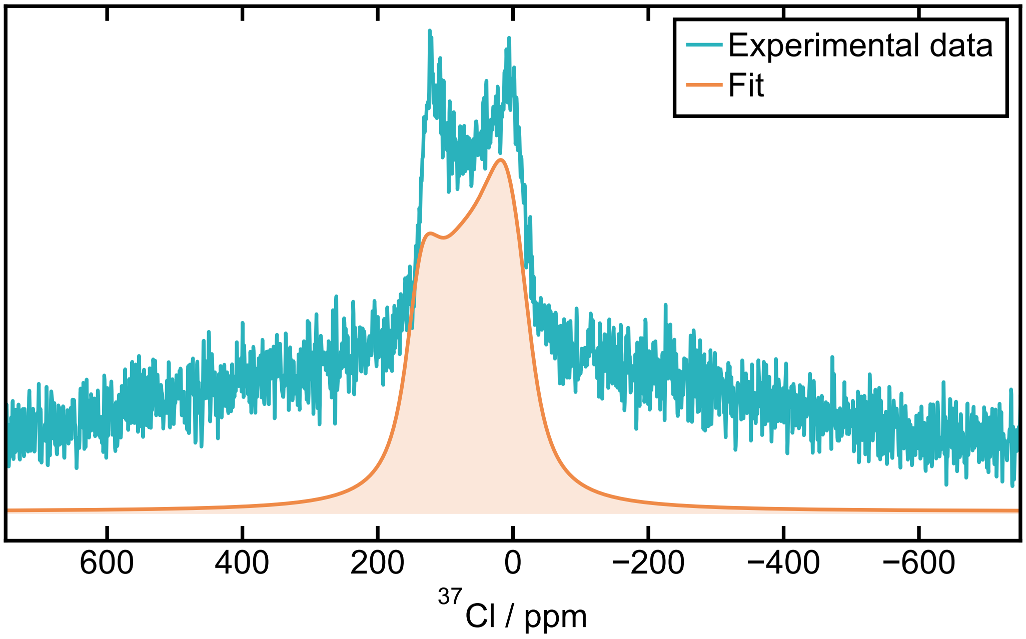

Figure S16: ^{37}Cl static Hahn echo of compound **2** fitted as a single site chlorine atom.

Table S8: Fitting parameters for ^{37}Cl static ssNMR of compound **2** obtained with ssNAKE.[5] Fit: Quadrupole+CSA, Units: ppm, I=3/2, MAS=Static, Satellites=False, Cheng=15, Angle=$arctan(\sqrt{2})$, Sidebands=32

C_q/ MHz	η	δ_{iso}/ ppm	Ω/ ppm	κ	Lorentz/ kHz	Gauss
1.66	0.25	90.21	12.62	-0.96	2.5	0.000

S4.3 Calculated chemical shifts

Table S9: Calculated chlorine chemical shifts for compound **2**.

Chlorines of interest	Isotropic Shielding Constants/ ppm	Chemical Shifts w.r.t. NaCl/ ppm	Quadrupolar Coupling Constants/ MHz
Cl in $Cl(H_2O)_6$	930.04	80.73	3.00
Cl in network	817.16	193.61	19.04
Cl in network	817.51	193.26	20.05
Cl in network	828.23	182.54	18.19
Cl in network	835.52	175.25	16.94
Cl in network	805.63	205.14	21.97
Cl in network	766.12	244.65	27.01
NaCl	1010.77		

S4.4 2D NMR of compound 2

In the ^{1}H-^{13}C CP-based 2D HCH experiment the spectral width of the indirect dimension was 13888 Hz, with a maximum t1 evolution time of 14.4 ms over 400 increments, recorded with 32 scans; the 13C transmitter frequency was set to 47.5 ppm. The ^{1}H-^{13}C CP step had a 67 kHz tangential shaped ^{1}H rf pulse and a rectangular 12 kHz rf pulse on 13C with a contact time of 2.5 ms.

In the ^{1}H-^{13}C 2D HSQC experiment the spectral width of the indirect dimension was 13888 Hz, with a maximum t1 evolution time of 14.4 ms over 400 increments, recorded with 32 scans; the 13C transmitter frequency was set to 30 ppm. The INEPT delay (1/4J) was set to 0.8 ms.

Figure S17 a) shows the ^{1}H-^{13}C CP-based 2D HCH spectrum and b) shows the ^{1}H-^{13}C 2D HSQC spectrum of compound **2**. These spectra show that the ^{1}H signals at 5.12 and 5.30 ppm are bound to carbon atoms. Thus, we assign these signals to the CH_2 groups of the DABCOnium cation.

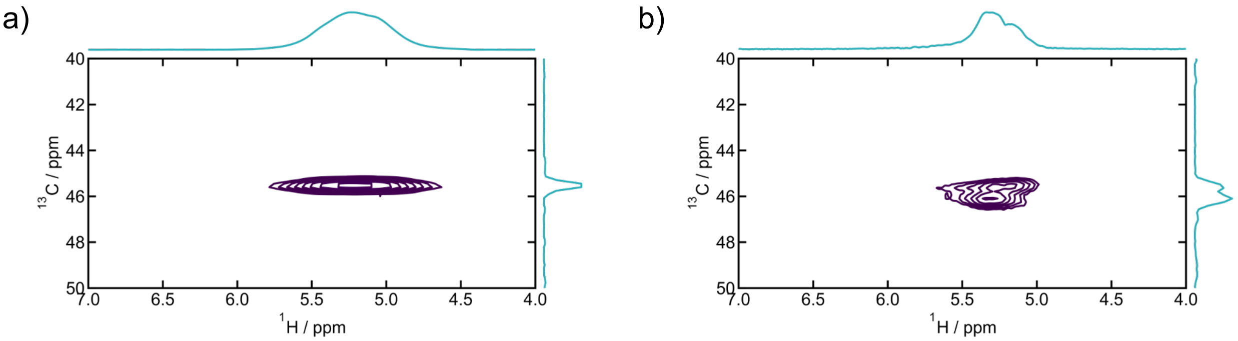

Figure S17: a) ^{1}H-^{13}C CP-based 2D HCH spectrum and b) ^{1}H-^{13}C 2D HSQC spectrum of compound **2** at 55 kHz and 0 °C.

In the ^{1}H-^{15}N CP-based 2D HNH experiment the spectral width of the indirect dimension was 1736 Hz, with a maximum t1 evolution time of 18.4 ms over 64 increments, recorded with 128 scans; the ^{15}N transmitter frequency was set to 36 ppm. The ^{1}H-^{15}N CP step had a 11 kHz tangential shaped ^{1}H rf pulse and a rectangular 44 kHz rf pulse on ^{15}N with a contact time of 150 us.

Figure S18 shows the ^{1}H-^{15}N CP-based 2D spectrum and ^{1}H-^{15}N HSQC MAS NMR of compound **2**. In the CP-based 2D spectrum, three environments are visible, while in the HSQC MAS NMR spectrum, four signals are visible. CP experiments work better for rigid molecules. The absence of one signal in the CP experiment compared to the HSQC experiment suggest that one NH group is rather mobile. We assign this mobile group to the NH group in proximity to the chloride hydrate cluster, and the proton is rather mobile and can exchange with the hydrate shell of the cluster.

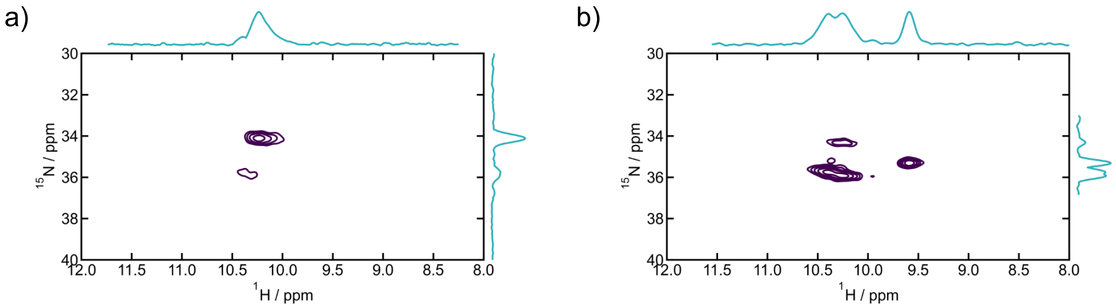

Figure S18: a) ^{1}H-^{15}N CP-based 2D spectrum and b) ^{1}H-^{15}N HSQC MAS NMR of compound **2** at 55 kHz and 0 °C.

Figure S19 shows the ^{1}H-^{1}H double quantum single quantum spectrum of compound **2**. The spectrum helps to identify protons that are close in space. The spectrum shows that the signals around 5 ppm are all in close proximity and thus belong to the CH_2 groups of DABCOnium. Furthermore, some of the protons of this signal are in proximity of the NH groups, suggesting they belong to the DABCOnium cation. Also, the signals of the water molecules of the chloride hydrate cluster have a chemical shift of 4 ppm and here it shows coupling with the protons of the NH groups. This confirms the assignment of the signal at 4 ppm to the hydrate shell of the cluster.

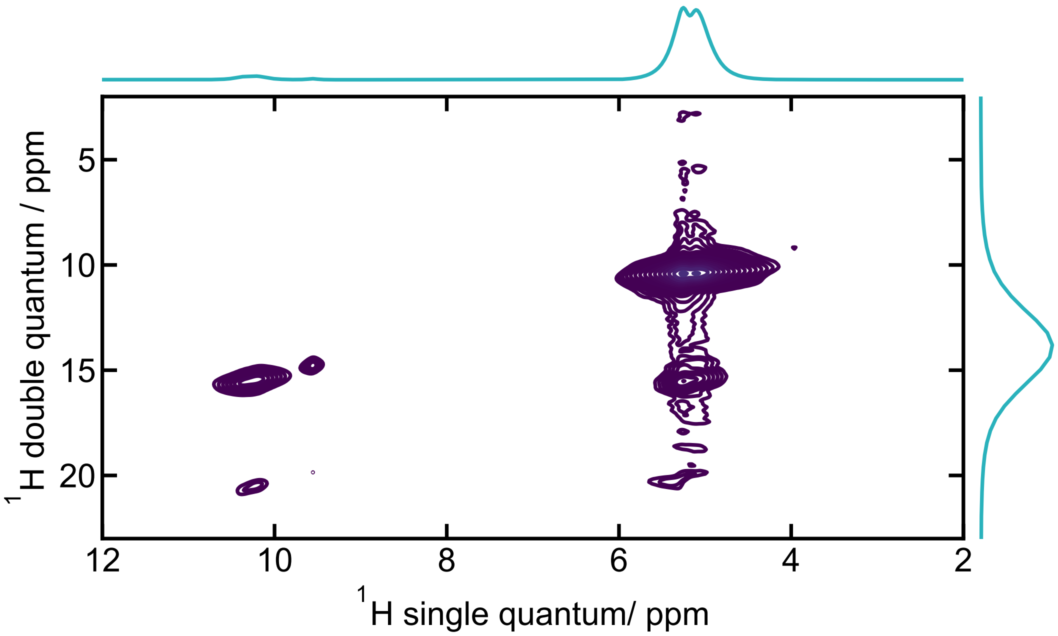

Figure S19: ^{1}H-^{1}H double quantum single quantum spectrum of compound **2** at 55 kHz and 0 °C.

S4.5 Temperature dependent ^{1}H NMR of compound 2

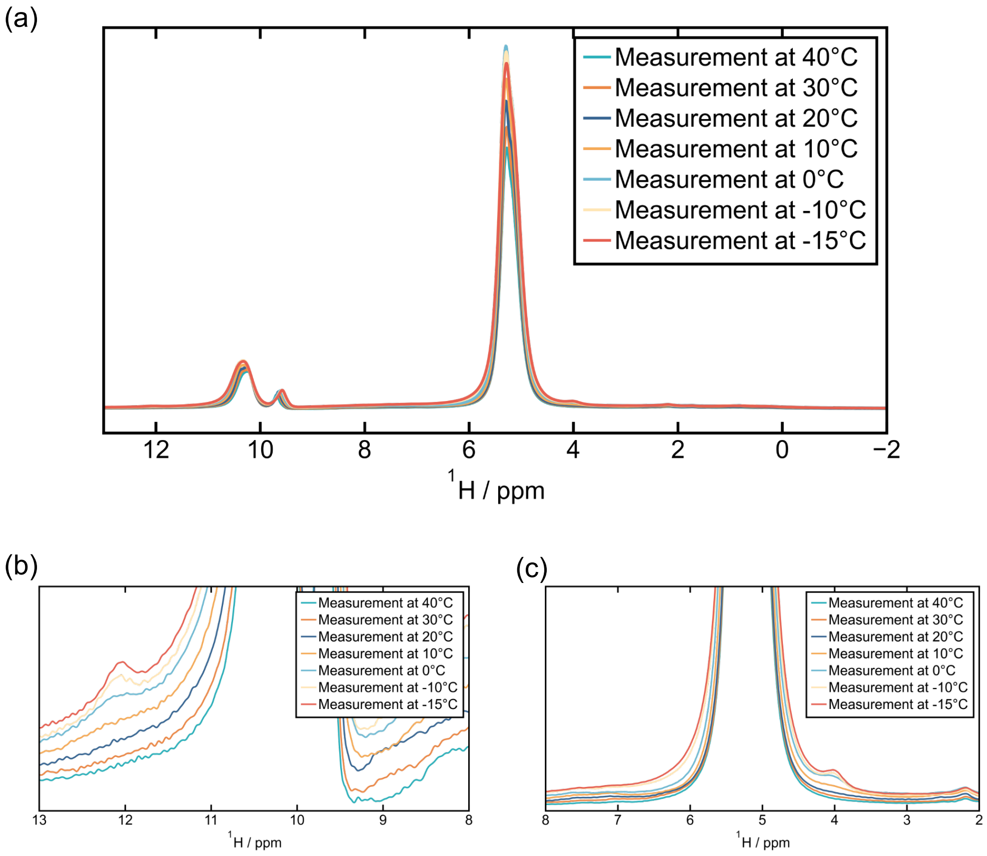

Figure S20: ^{1}H NMR spectrum of **2** measured in the temperature range from -15 to 40 °C. a) Full spectrum, b) Close-up of the region of NH signals, c) Close-up of the region of CH and H_2O signals.

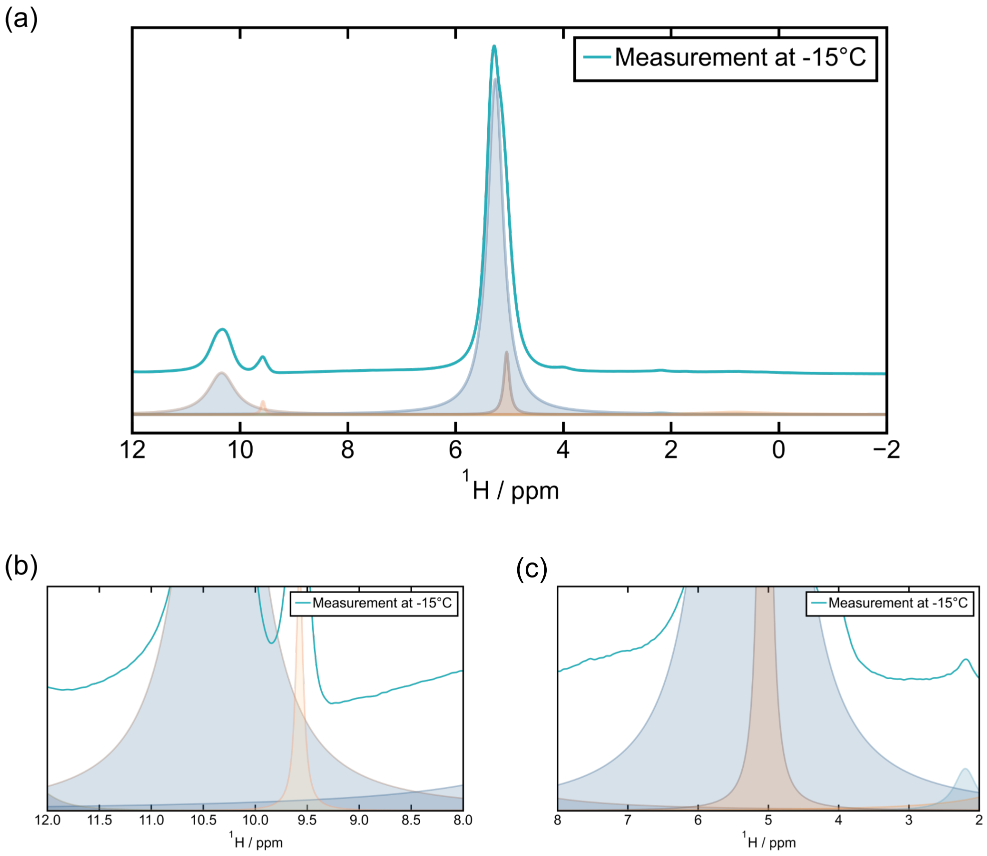

Figure S21: ^{1}H NMR spectrum of **2** measured at $-15\,°C$. a) Full spectrum, b) Close-up of the region of NH signals, c) Close-up of the region of CH and H_2O signals.

Table S10: Fitting parameters for ^{1}H MAS ssNMR at $-15\,°C$ obtained with *ssNAKE*.[5]

Position/ ppm	Integral	Lorentz
12.14	1.606e+11	311.0
10.34	6.837e+12	419.3
9.57	2.972e+11	57.20
5.26	3.239e+13	247.1
5.05	2.106e+12	86.14
2.19	2.352e+11	264.9
1.73	3.274e+10	114.1
0.80	1.054e+12	1097.0

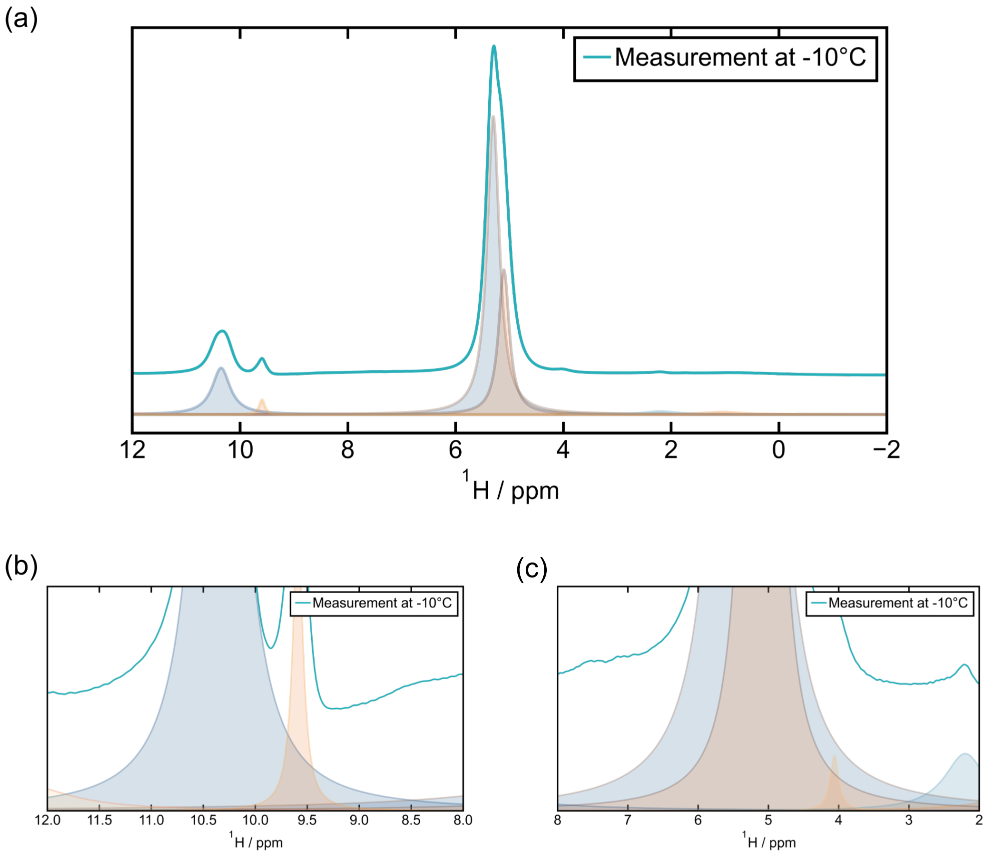

Figure S22: ^{1}H NMR spectrum of **2** measured at −10 °C. a) Full spectrum, b) Close-up of the region of NH signals, c) Close-up of the region of CH and H_2O signals.

Table S11: Fitting parameters for ^{1}H MAS ssNMR at $-10\,^{\circ}$C obtained with *ssNAKE*.[5]

Position/ ppm	Integral	Lorentz
12.25	4.265e+11	770.7
10.35	5.334e+12	284.4
9.59	5.011e+11	84.29
5.30	2.323e+13	193.5
5.10	9.869e+12	169.3
4.06	1.205e+11	104.1
2.20	6.051e+11	503.2
0.10	6.124e+11	615.1

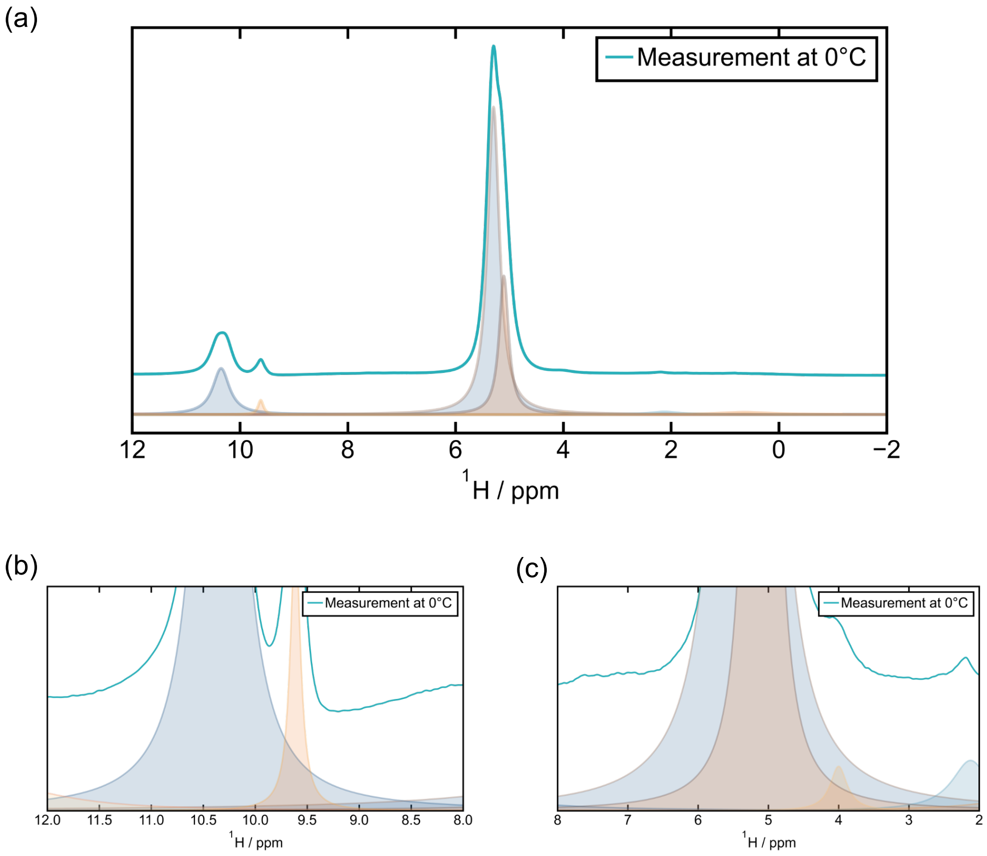

Figure S23: ^{1}H NMR spectrum of **2** measured at 0 °C. a) Full spectrum, b) Close-up of the region of NH signals, c) Close-up of the region of CH and H_2O signals.

Table S12: Fitting parameters for ^{1}H MAS ssNMR at 0 °C obtained with *ssNAKE*.[5]

Position/ ppm	Integral	Lorentz
12.42	5.024e+11	1003.
10.35	4.958e+12	263.1
9.61	4.819e+11	82.52
5.30	2.342e+13	186.6
5.10	8.313e+12	146.9
4.00	1.916e+11	204.9
2.12	4.801e+11	453.7
0.64	8.083e+11	825.1

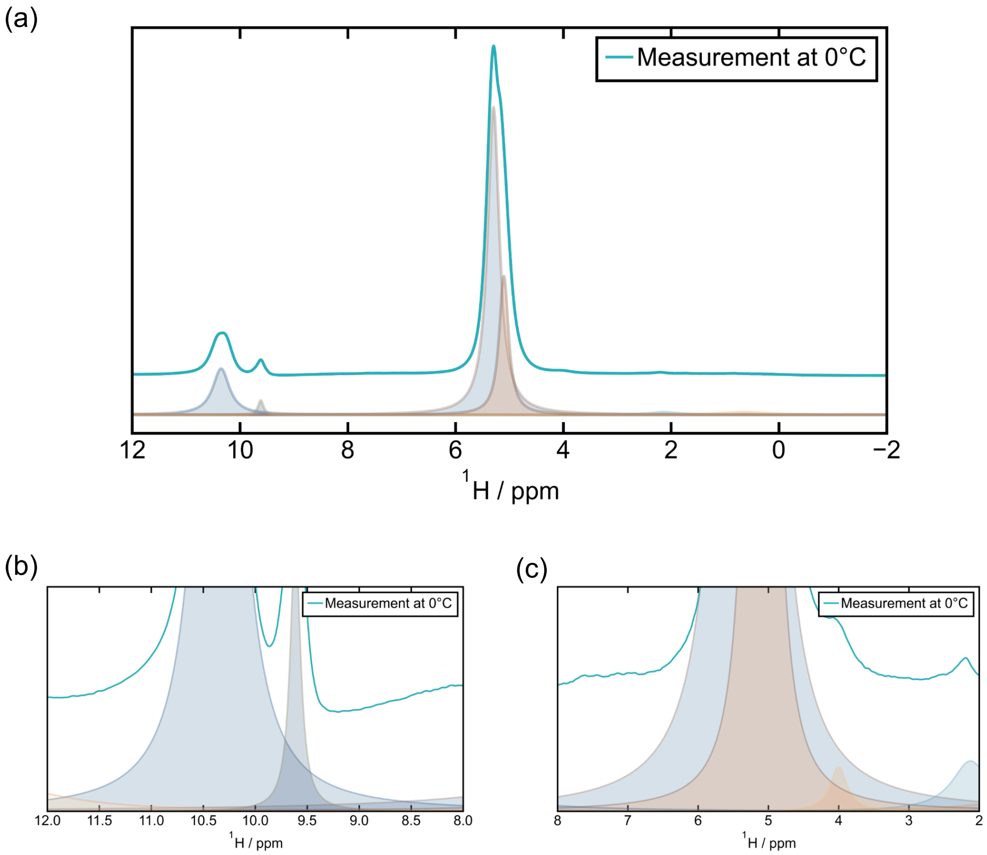

Figure S24: ^{1}H NMR spectrum of **2** measured at 10 °C. a) Full spectrum, b) Close-up of the region of NH signals, c) Close-up of the region of CH and H_2O signals.

Table S13: Fitting parameters for ^{1}H MAS ssNMR at 10 °C obtained with *ssNAKE*.[5]

Position/ ppm	Integral	Lorentz
10.46	6.139e+11	144.2
10.30	3.698e+12	237.0
9.63	4.366e+11	80.73
5.30	1.812e+13	167.2
5.12	9.854e+12	162.1
2.13	8.710e+11	897.2
0.68	6.297e+11	845.9

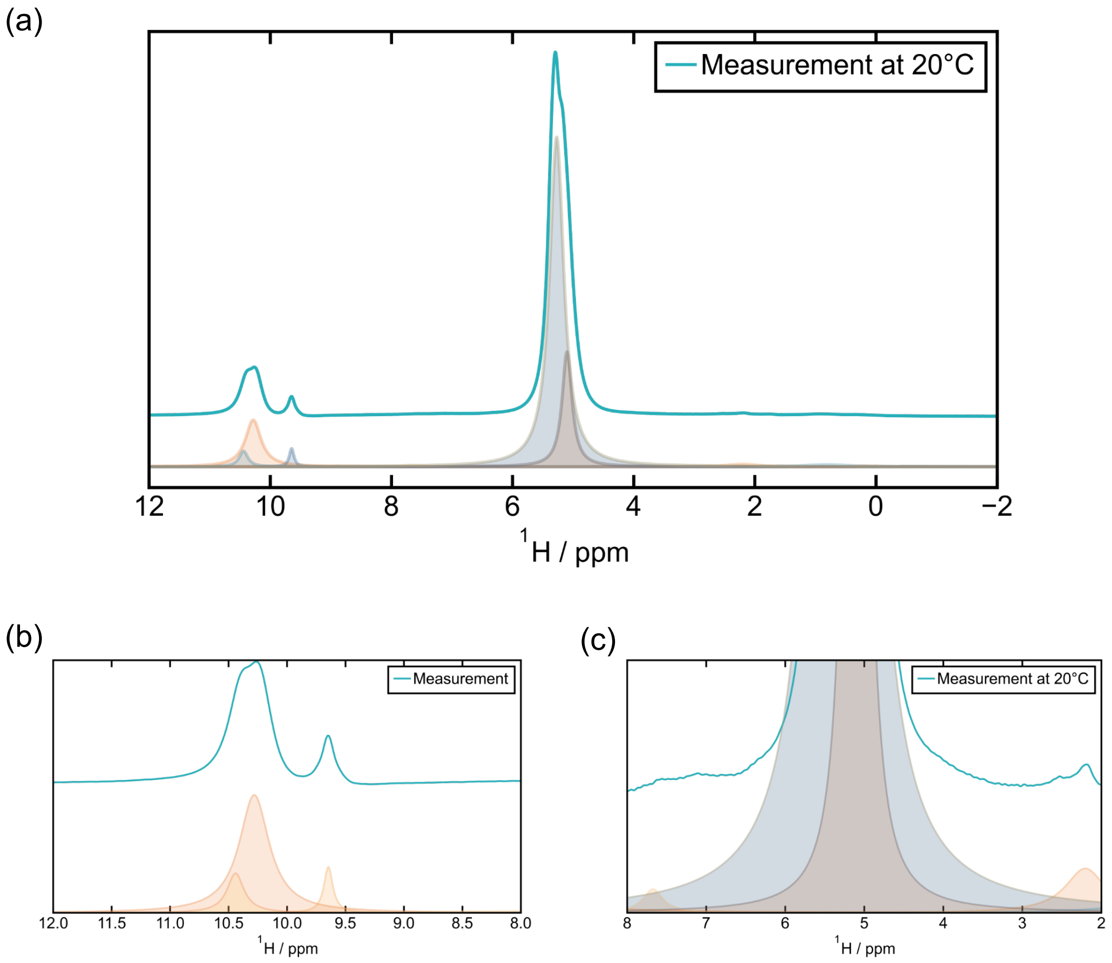

Figure S25: ^{1}H NMR spectrum of **2** measured at 20 °C. a) Full spectrum, b) Close-up of the region of NH signals, c) Close-up of the region of CH and H_2O signals.

Table S14: Fitting parameters for ^{1}H MAS ssNMR at 20 °C obtained with *ssNAKE*.[5]

Position/ ppm	Integral	Lorentz
10.44	6.022e+11	122.5
10.28	3.206e+12	218.7
9.65	3.703e+11	64.65
7.67	9.623e+10	219.0
5.27	2.072e+13	200.4
2.20	3.582e+11	432.8
0.82	4.366e+11	622.2
0.15	7.359e+10	300.6

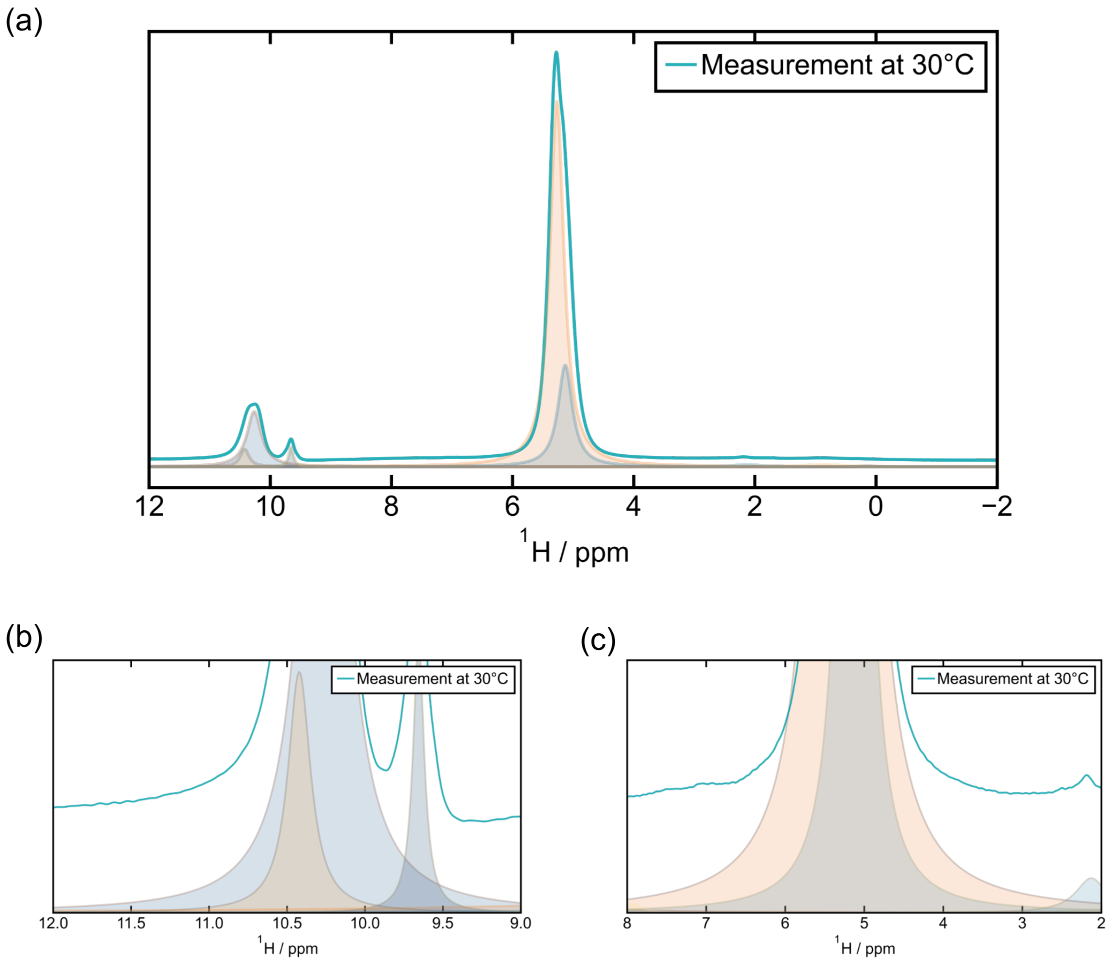

Figure S26: ^{1}H NMR spectrum of **2** measured at 30 °C. a) Full spectrum, b) Close-up of the region of NH signals, c) Close-up of the region of CH and H_2O signals.

Table S15: Fitting parameters for ^{1}H MAS ssNMR at 30 °C obtained with *ssNAKE*.[5]

Position/ ppm	Integral	Lorentz
10.41	5.542e+11	123.2
10.72	2.868e+12	203.4
9.66	3.138e+11	62.16
7.94	3.215e+10	200.0
5.27	1.848e+13	197.6
5.13	5.289e+12	203.4
2.12	2.177e+11	336.6
0.86	3.285e+11	579.4
0.16	4.142e+10	232.5

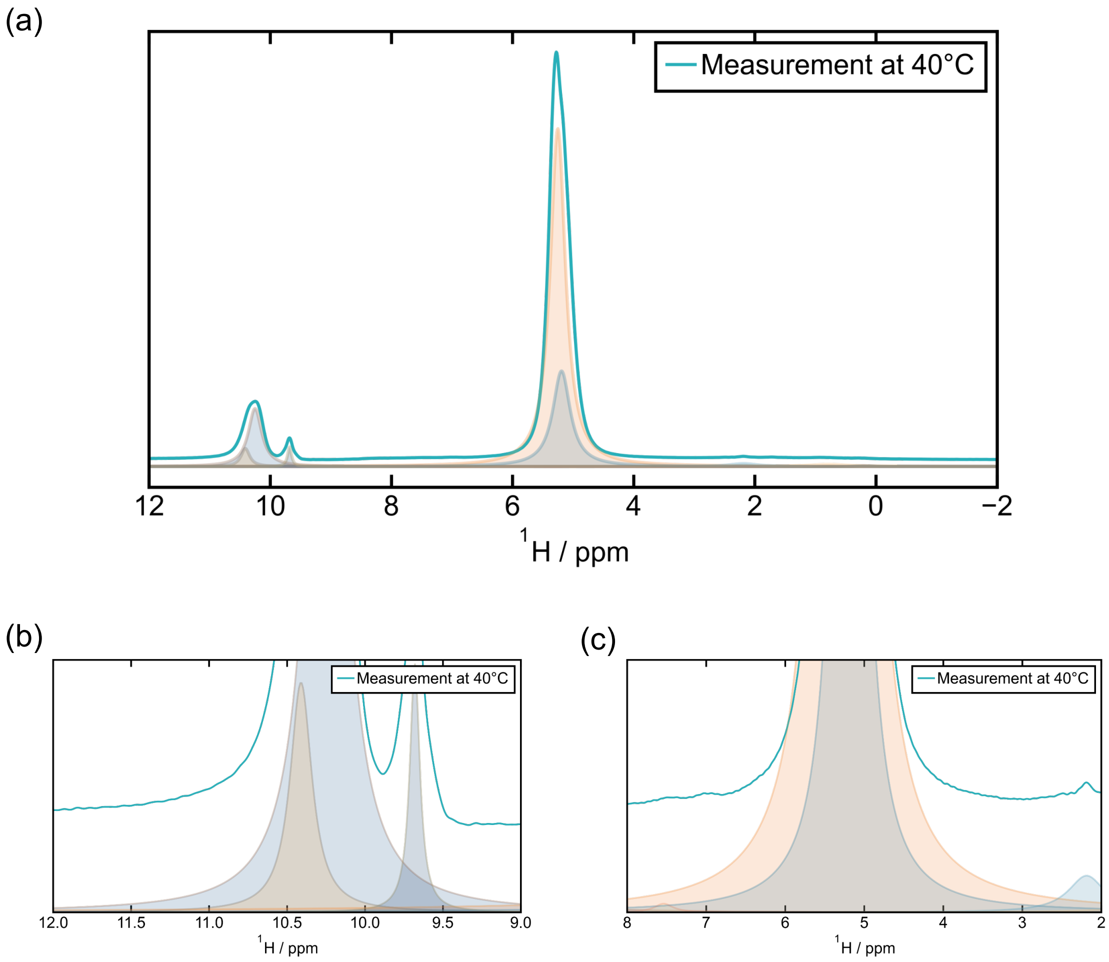

Figure S27: ^{1}H NMR spectrum of **2** measured at 40 °C. a) Full spectrum, b) Close-up of the region of NH signals, c) Close-up of the region of CH and H_2O signals.

Table S16: Fitting parameters for ^{1}H MAS ssNMR at 40 °C obtained with *ssNAKE*.[5]

Position/ ppm	Integral	Lorentz
10.41	5.111e+11	119.1
10.25	2.571e+12	186.8
9.68	2.544e+11	54.93
7.54	3.215e+10	200.0
5.25	1.719e+13	214.2
5.19	5.540e+12	244.1
2.19	2.699e+11	395.1
0.78	2.997e+11	574.9
0.20	4.142e+10	232.5

S4.6 Solid state NMR of $(DABCOH_2)_4Sb^{III}{}_2Cu^{II}{}_2Cl_{18}(H_2O)_4$

Fitting parameters for ^{37}Cl MAS ssNMR and ^{37}Cl static ssNMR

The static signal was analysed by keeping C_Q, η and δ_{iso} fixed as determined from the MAS sample.

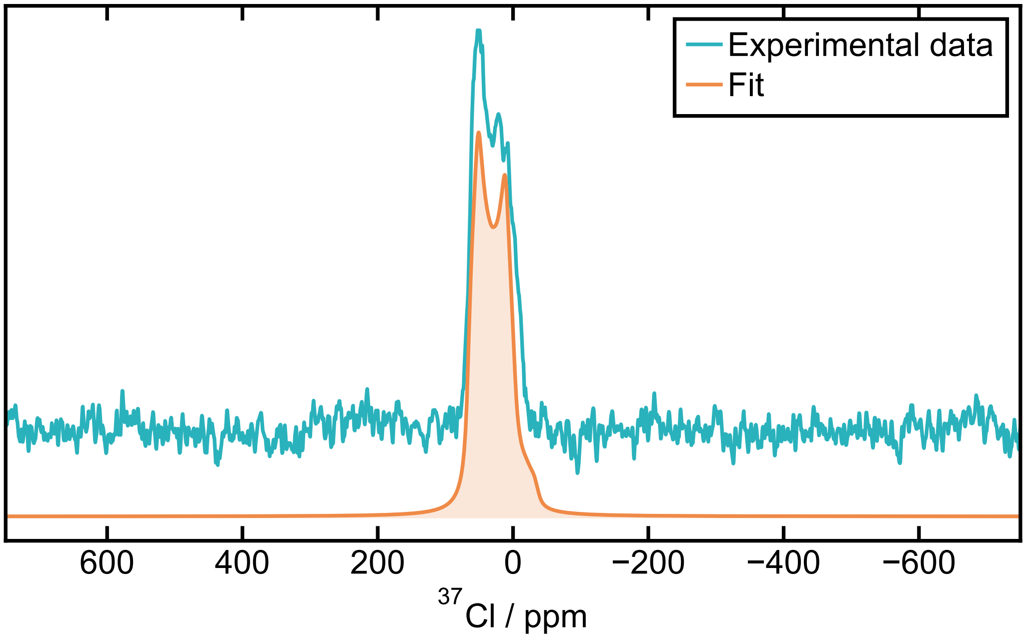

Figure S28: ^{37}Cl MAS ssNMR of $(DABCOH_2)_4Sb^{III}{}_2Cu^{II}{}_2Cl_{18}(H_2O)_4$ fitted as a single site chlorine atom.

Table S17: Fitting parameters for ^{37}Cl MAS ssNMR of $(DABCOH_2)_4Sb^{III}{}_2Cu^{II}{}_2Cl_{18}(H_2O)_4$ obtained with ssNAKE.[5] Fit: Quadrupole, Units: ppm, I=3/2, MAS=Finite MAS, Satellites=False, Cheng=15, Angle=$arctan(\sqrt{2})$, Sidebands=32

$\delta_{iso}/\ ppm$	$C_q/\ MHz$	η	Lorentz/ kHz	Gauss	LorentzST
74.73	2.66	0.072	0.9	0.00	1.00

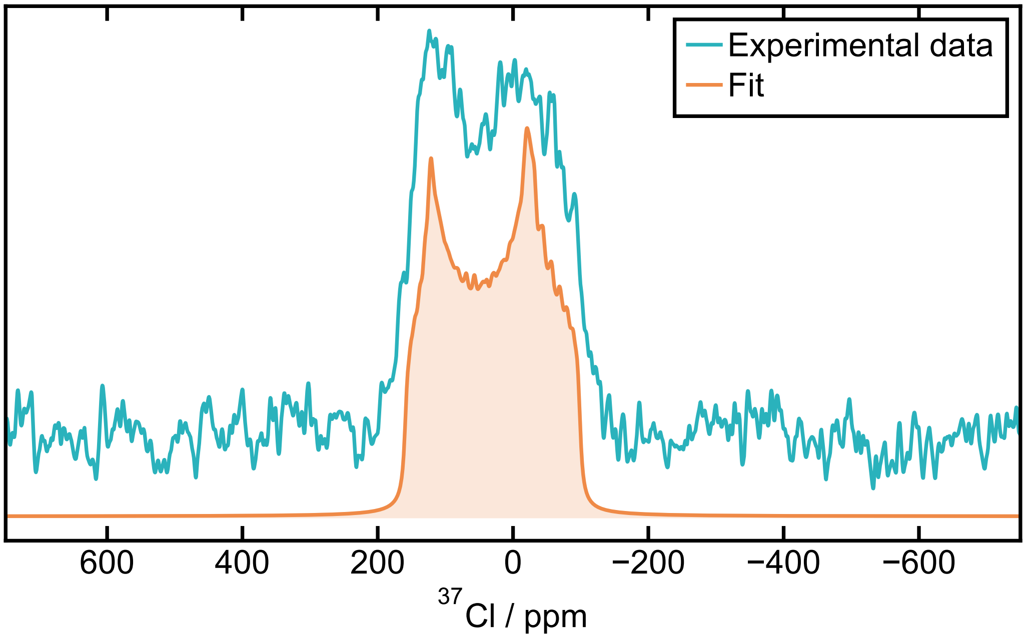

Figure S29: ^{37}Cl static Hahn echo of $(DABCOH_2)_4Sb^{III}{}_2Cu^{II}{}_2Cl_{18}(H_2O)_4$ fitted as a single site chlorine atom.

Table S18: Fitting parameters for ^{37}Cl static ssNMR of $(DABCOH_2)_4Sb^{III}{}_2Cu^{II}{}_2Cl_{18}(H_2O)_4$ obtained with ssNAKE.[5] Fit: Quadrupole+CSA, Units: ppm, I=3/2, MAS=Static, Satellites=False, Cheng=15, Angle=$arctan(\sqrt{2})$, Sidebands=32

C_q/ MHz	η	δ_{iso}/ ppm	Ω/ ppm	κ	Lorentz/ kHz	Gauss
2.66	0.072	74.73	-80.75	0.68	1.6	0.00

Fitting parameters for ^{35}Cl MAS ssNMR and ^{35}Cl static ssNMR

Table S19: Fitting parameters for ^{35}Cl MAS ssNMR of $(DABCOH_2)_4Sb^{III}{}_2Cu^{II}{}_2Cl_{18}(H_2O)_4$ obtained with ssNAKE.[5] Fit: Quadrupole, Units: ppm, I=3/2, MAS=Finite MAS, Satellites=False, Cheng=15, Angle=$arctan(\sqrt{2})$, Sidebands=32

δ_{iso}/ *ppm*	C_q/ *MHz*	η	Lorentz/ *kHz*	Gauss	LorentzST
75.21	2.06	0.23	0.5	0.00	1.00

Table S20: Fitting parameters for ^{35}Cl static ssNMR of $(DABCOH_2)_4Sb^{III}{}_2Cu^{II}{}_2Cl_{18}(H_2O)_4$ obtained with ssNAKE.[5] Fit: Quadrupole+CSA, Units: ppm, I=3/2, MAS=Static, Satellites=False, Cheng=15, Angle=$arctan(\sqrt{2})$, Sidebands=32

C_q/ *MHz*	η	δ_{iso}/ *ppm*	Ω/ ppm	κ	Lorentz/ *kHz*	Gauss
2.06	0.23	75.21	-60.87	0.59	0.4	0.000

S5 Optical spectroscopy

S5.1 Compound 1

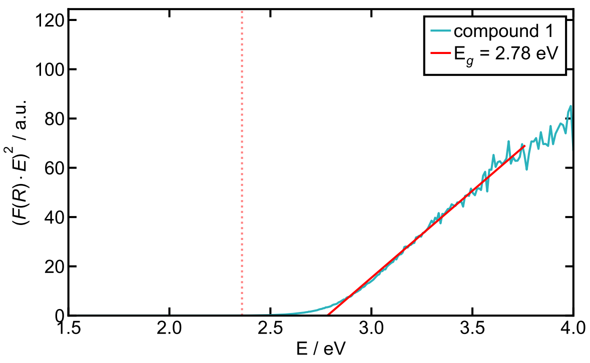

Figure S30: Tauc plot of compound **1** assuming a direct allowed transition.

S5.2 Compound 2

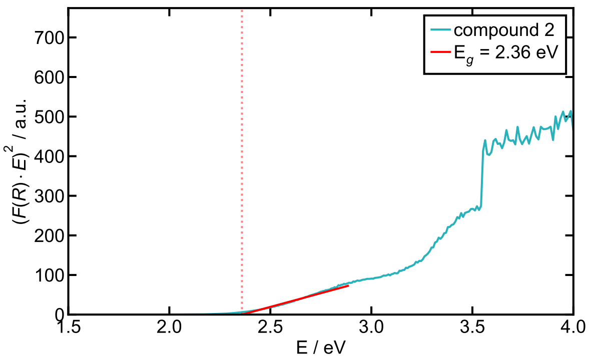

Figure S31: Tauc plot of compound **2** assuming a direct allowed transition.

S6 Infrared spectroscopy

S6.1 Compound 1

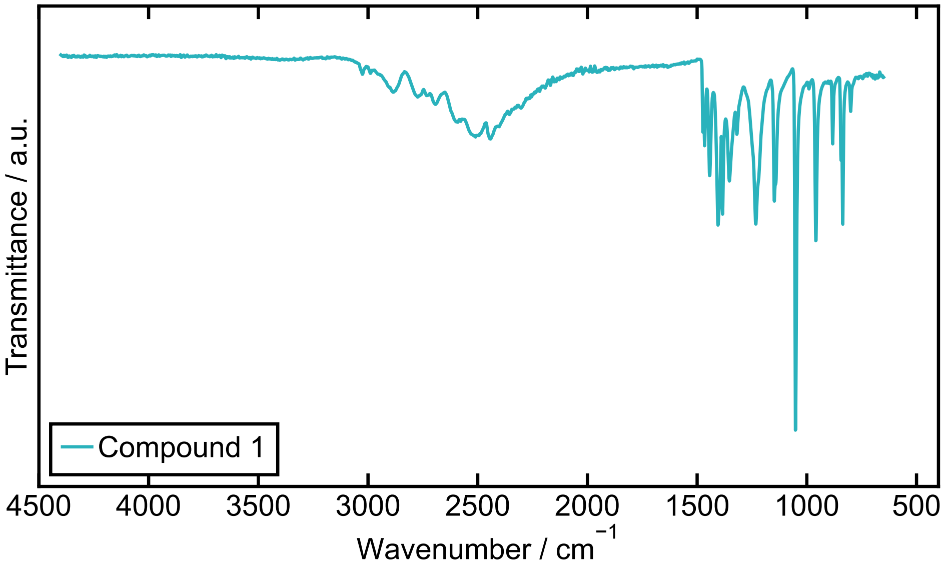

Figure S32: FT-IR spectrum of compound **1**.

S6.2 Compound 2

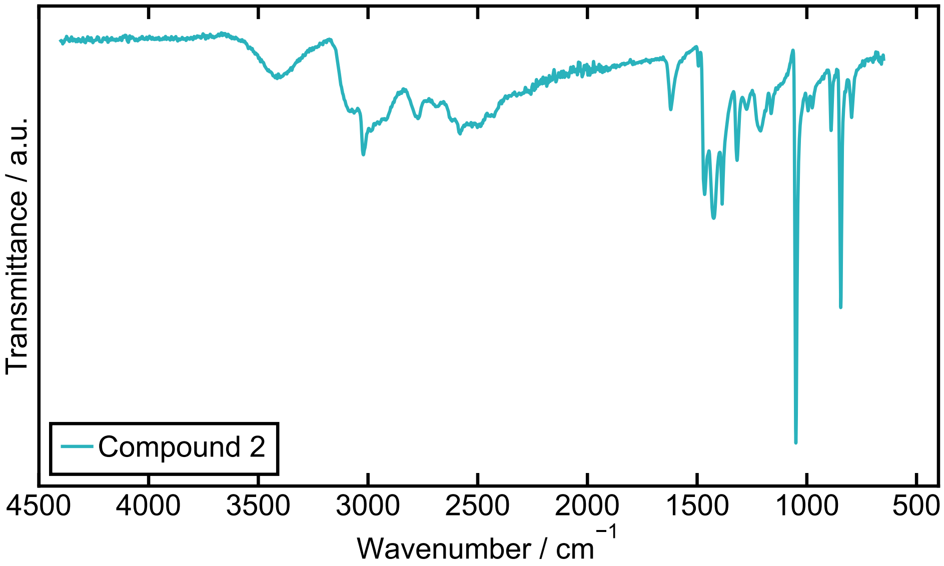

Figure S33: FT-IR spectrum of compound **2**.

S7 Simultaneous Thermal Analysis (STA)

Simultaneous Thermal Analysis (STA). STA measurements were obtained with a STA449 F5 Jupiter instrument by Netzsch. Corundum crucibles were filled with the sample. They were heated dynamically with heating rates of 10 K min^{-1} under Ar flow. The data were analyzed with the Netzsch Proteus 61 software package.

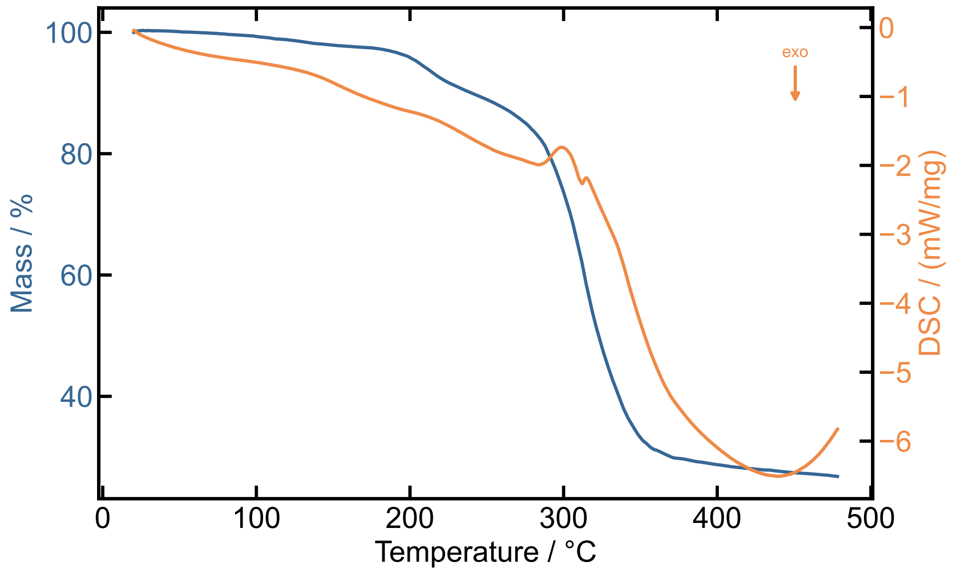

Figure S34: Simultaneous thermal analysis of compound **2**. The material loses 3 wt% up to 175 °C.

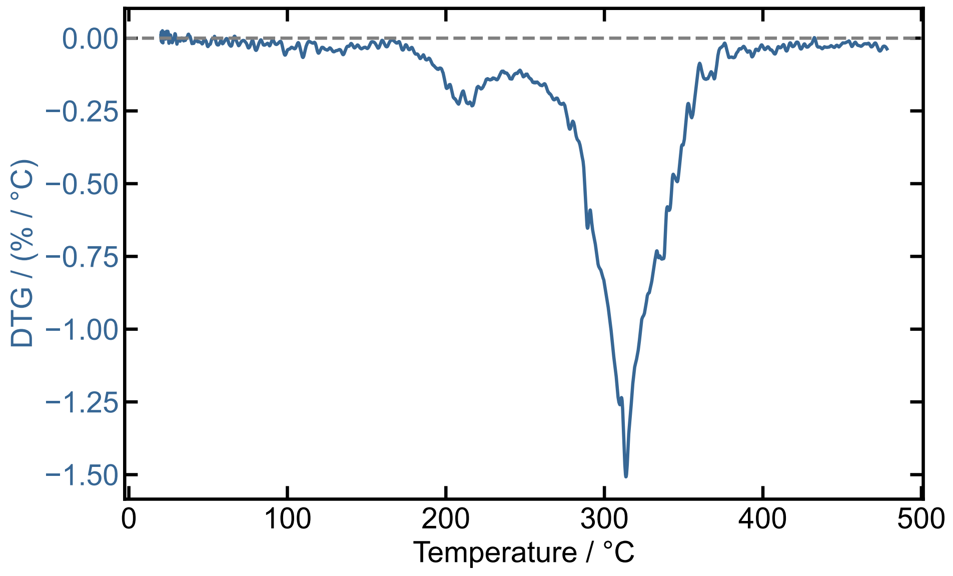

Figure S35: Derivative thermogravimetric (DTG) curve of compound **2**.

References

(1) Orosel, D.; Balog, P.; Liu, H.; Qian, J.; Jansen, M. Sb_2O_4 at High Pressures and High Temperatures. *J. Solid State Chem.* **2005**, *178*, 2602–2607.

(2) Atkinson, L.; Day, P. Charge transfer in mixed-valence solids. Part IV. Electronic spectra of hexachloroantimonates(III, V). *J. Chem. Soc. A* **1969**, 2423.

(3) Benin, B. M.; McCall, K. M.; Wörle, M.; Borgeaud, D.; Vonderach, T.; Sakhatskyi, K.; Yakunin, S.; Günther, D.; Kovalenko, M. V. Lone-Pair-Induced Structural Ordering in the Mixed-Valent 0D Metal-Halides $Rb_{23}Bi_x^{III}Sb_{7-x}^{III}Sb_2^VCl_{54}$ ($0 \leq x \leq 7$). *Chem. Mater.* **2021**, *33*, 2408–2419.

(4) Liu, Y.; Liang, J.; Deng, Z.; Guo, S.; Ji, X.; Chen, C.; Canepa, P.; Lü, X.; Mao, L. 0D Pyramid-intercalated 2D Bimetallic Halides with Tunable Electronic Structures and Enhanced Emission under Pressure. *Angew. Chem. Int. Ed.* **2023**, *62*, e202314977.

(5) Van Meerten, S.; Franssen, W.; Kentgens, A. ssNake: A cross-platform open-source NMR data processing and fitting application. *J. Magn. Reson.* **2019**, *301*, 56–66.
